# CXCL14 Promotes Skeletal Muscle Mass Growth and Attenuates Lipopolysaccharide‐ and Dexamethasone‐Induced Muscle Atrophy in Cultured Myotubes and Mouse Models

**DOI:** 10.1002/jcsm.70087

**Published:** 2025-10-14

**Authors:** Bagus Sarmito, Younjeong Oh, Nurkyz Alymkulova, Jeong Kyo Yoon

**Affiliations:** ^1^ Department of Integrated Biomedical Science, Graduate School Soonchunhyang University Cheonan South Korea; ^2^ Soonchunhyang Institute of Medi‐Bio Science Soonchunhyang University Cheonan South Korea

**Keywords:** CXCL14, protein metabolism, skeletal muscle atrophy, skeletal muscle homeostasis, skeletal muscle hypertrophy

## Abstract

**Background:**

Skeletal muscle mass is regulated by secretory factors derived from myofibers and muscle‐resident cells. Identifying these factors and understanding their mechanisms is critical for combating muscle wasting disorders. This experimental study investigates the role of CXCL14, a chemokine primarily secreted by fibro‐adipogenic progenitors (FAPs) residing in muscle, in regulating muscle mass.

**Methods:**

This study was conducted at the Soonchunhyang Institute of Medi‐bio Science (SIMS), South Korea, between August 2020 and June 2025. Mouse C2C12 myotubes and primary human myotubes were treated with recombinant CXCL14, with or without co‐treatment using *Rps6kb1* siRNA, lipopolysaccharide (LPS) or dexamethasone (DEX). Myotube mass index (MMI) was measured. Expression of AKT‐S6 kinase (S6K), FOXO‐Atrogin‐1/MuRF‐1 signalling components and myosin heavy chains (MyHCs) was assessed via Western blotting. Eight‐week‐old male mice were used: ICR mice for electroporation experiments and C57BL/6N strain for LPS and DEX atrophy models. *Cxcl14* expression plasmids were electroporated into tibialis anterior (TA) muscles, with or without LPS or DEX treatment. Cross‐sectional area (CSA) of myofibers was measured; Western blotting and RNA sequencing were used to analyse molecular responses. Statistical analyses included one‐way ANOVA with Tukey's post hoc test, repeated‐measures ANOVA with Dunnett's post hoc test, Kruskal–Wallis test with Dunn's post hoc test and unpaired Student's *t*‐test, as appropriate.

**Results:**

CXCL14 induced hypertrophy in C2C12‐derived myotubes: (MMI [μm^2^]: 100 ng/mL CXCL14, 1345 ± 50.97 [95% CI: 1237–1453], vs. control, 897.9 ± 33.33 [95% CI: 829.8–996], *p ≤* 0.0001). *Cxcl14* overexpression in mouse TA muscles significantly increased muscle mass: (CSA [μm^2^]: HA‐CXCL14: 1408 ± 15.42 [95% CI: 1378–1438]; CXCL14‐Myc: 1499 ± 17.18 [95% CI: 1464–1534]; control: 870.1 ± 11.25 [95% CI: 848.1–892.2], *p ≤* 0.0001). CXCL14 activated the AKT‐S6K pathway and inhibited the FOXO‐Atrogin‐1/MuRF‐1 pathway in both in vitro and in vivo models. CXCL14 effectively reversed LPS‐ and DEX‐induced atrophy in both C2C12 myotubes and TA muscles, as demonstrated by corresponding increases MMI and CSA (all *p* ≤ 0.0001). CXCL14 also promoted hypertrophy in primary human myotubes in vitro (MMI [μm^2^]: 100 ng/mL CXCL14, 3481 ± 242.6 [95% CI: 2973–3989] vs. control, 2549 ± 114.7 [95% CI: 2310–2787], *p* ≤ 0.001) and significantly reversed atrophy induced by LPS and DEX (*p* ≤ 0.01 to *p* ≤ 0.0001), accompanying the activation of protein synthesis and inhibition of protein degradation pathways.

**Conclusions:**

Our findings identify CXCL14 as a novel regulator of skeletal muscle mass and highlight its therapeutic potential in preventing or reversing muscle atrophy associated with ageing and diseases in humans.

## 1 Introduction

Skeletal muscle plays a pivotal role not only in voluntary movement and posture but also in systematic metabolic regulation. Maintaining muscle mass and function is essential for preserving motility, metabolic health and overall quality of life, particularly in ageing populations and individuals with chronic diseases. Muscle mass is largely maintained by a balance between protein synthesis and degradation in myofibers, a process regulated by key molecular pathways such as AKT‐S6 Kinase (S6K) and the ubiquitin‐proteasome system [1]. While the AKT‐S6K pathway promotes anabolic signalling and muscle growth, disruptions to this balance—due to ageing, disuse, inflammation or disease—can lead to muscle atrophy, frailty and metabolic disturbances.

Chemokines, a family of small cytokines, are traditionally known for orchestrating immune cell migration during inflammation and immune responses [2]. However, recent studies have identified their implicating roles in skeletal muscle homeostasis and regeneration. For instance, CCL2 (monocyte chemoattractant protein‐1) plays a critical role in the inflammatory response following muscle injury [3]. It attracts macrophages to the injury site, where proinflammatory M1 macrophages initiate debris clearance, and anti‐inflammatory M2 macrophages secrete growth factors that support muscle repair. Another chemokine, CXCL12 (stromal cell‐derived factor‐1, SDF‐1), facilitates the recruitment and activation of satellite cells—the resident muscle stem cells responsible for muscle regeneration [4]. In contrast, CXCL14 has been shown to negatively regulate myogenesis [5]. *Cxcl14* gene knockdown enhances myogenic differentiation and cell fusion in a cell autonomous manner in vitro and accelerates muscle regeneration.

Chemokines also play a pivotal role in the regulation of myofiber mass, a process highly relevant to muscle wasting conditions. Elevated levels of CCL2 and CXCL8 are frequently observed in conditions such as sarcopenia and cachexia [6, 7, 8]. Persistent activation of their signalling pathways promotes immune cell infiltration, drives chronic inflammation and enhances catabolic processes and muscle breakdown through activation of the ubiquitin‐proteasome and autophagy‐lysosome systems [9]. Notably, CCL2 demonstrates dual, context‐dependent roles. While intramuscular administration of CCL2 reduces quadriceps muscle mass in healthy mice [10], it paradoxically protects against dexamethasone (DEX)‐induced muscle atrophy in vitro [11], highlighting the complexity of chemokine signalling in muscle biology.

Recent single‐cell transcriptome analyses revealed that *Cxcl14* is abundantly expressed in fibro‐adipogenic progenitors (FAPs), which reside in the interstitial space between myofibers in skeletal muscles [12]. Interestingly, ablation of FAPs in uninjured muscles leads to profound atrophy [13], likely due to the loss of key trophic factors they secrete, such as interleukin (IL)‐6 [14], IL‐10 [14] and CXCL14 [12] that can positively regulate skeletal muscle mass. While CXCL14 has a negative role in myogenesis [5], CXCL14 influences insulin sensitivity and glucose uptake in skeletal muscle cells [15], indicating a potential role in maintaining metabolic homeostasis within the muscle. However, the functional role of CXCL14 in skeletal muscle mass regulation under physiological or pathological conditions remains poorly understood.

Here, we demonstrate that CXCL14 acts as a novel, positive regulator of skeletal muscle mass, activating the AKT‐S6K signalling pathway while suppressing FOXO transcription factors—thus tipping the metabolic balance towards protein synthesis and hypertrophy. In both in vitro and in vivo models, CXCL14 attenuated muscle atrophy induced by lipopolysaccharide (LPS) and dexamethasone (DEX), agents that mimic inflammation—and steroid‐induced muscle wasting, respectively. Transcriptomic analysis further revealed that CXCL14 enhances CCL2‐CCR2 signalling while downregulating key atrophy‐associated genes like *Mstn* (*myostatin*) and *Trim63* (*Murf‐1*).

In summary, this study uncovers a previously uncharacterized role for CXCL14 in skeletal muscle mass regulation and establishes its potential as a therapeutic target for muscle atrophy. Our findings have important implications in the context of sarcopenia, cachexia and other muscle wasting disorders commonly observed in chronic diseases and ageing.

## 2 Materials and Methods

This laboratory‐based study including both in vitro and in vivo experiments was designed to investigate the role of CXCL14 in skeletal muscle mass regulation. All experiments were conducted at the Soonchunhyang Institute of Medi‐bio Science, South Korea, between August 2020 and June 2025.

### 2.1 Cell Culture and Mice

The C2C12 mouse myoblast cell line and the primary human skeletal myoblast (hSkM) were obtained from the American Type Culture Collection and Thermo Fisher (Gibco, A12555), respectively, and were maintained in culture following the providers' protocols. All mouse experiments were approved by the Soonchunhyang University Animal Care and Use Committee. Mice were housed in a specific pathogen‐free facility in the Soonchunhyang Institute of Medi‐bio Science on a 12‐h light/dark cycle and fed a standard chow diet. Eight‐week‐old ICR male mice (ORIENT Bio, South Korea) were used for in vivo plasmid DNA electroporation in tibialis anterior (TA) muscles. In atrophy model experiments, 8‐week‐old C57BL6/N male mice (ORIENT Bio, South Korea) were used.

### 2.2 Statistical Analysis

Experiments were conducted in duplicate or triplicate as indicated. Data are the mean ± standard error of the mean (SEM) and analysed with GraphPad Prism Version 9.0 (GraphPad Software, USA) unless specified in the figure captions. The *p* values were calculated by one‐way analysis of variance (ANOVA) with Tukey's post hoc test, repeated measures ANOVA with Dunnett's post hoc test, Kruskal–Wallis with Dunn's post hoc test or unpaired Student's *t*‐test as indicated. Statistical significance is indicated as *p ≤ 0.05, **p ≤ 0.01, ***p ≤ 0.001 or ****p ≤ 0.0001.

### 2.3 Additional Methods

Additional information regarding the materials and methods can be found in the Supporting Information.

## 3 Results

### 3.1 CXCL14 Activates the AKT‐S6K Signalling Pathway, Leading to Increased Protein Synthesis and Hypertrophy in Myotubes

**FIGURE 1:**
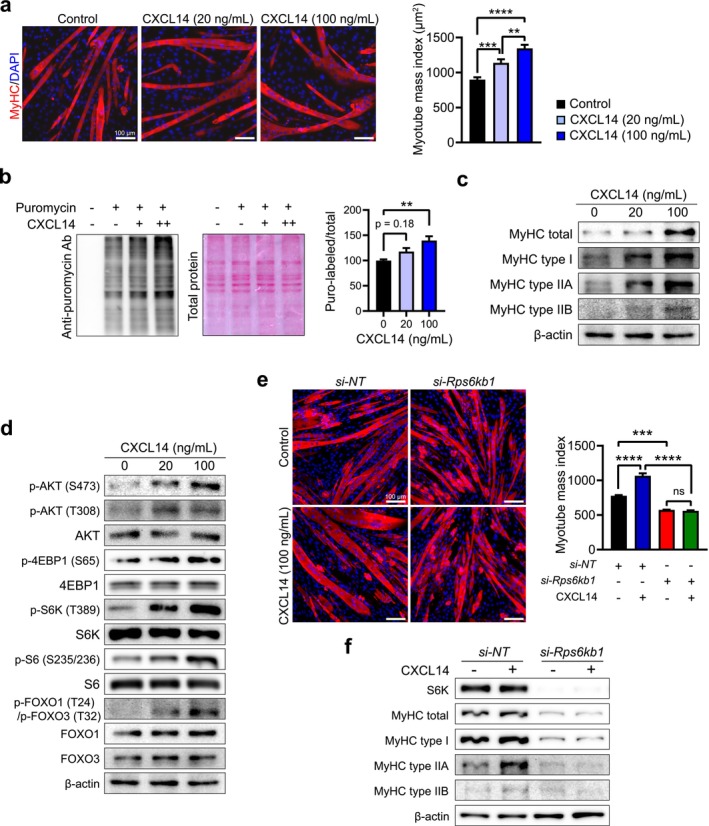
CXCL14 promotes myotube hypertrophy through activation of the AKT‐S6K pathway and suppression of the FOXO‐Atrogin‐1/MuRF‐1 pathway. (a) Representative immunofluorescence images of myotubes treated with CXCL14. C2C12 cell‐derived myotubes were treated with recombinant mouse CXCL14 protein (20 and 100 ng/mL) for 2 days in growth medium. Myotubes were stained with anti‐MyHC antibody (red) and 4′,6‐diamidino‐2‐phenylindole (DAPI; blue) for nuclei counterstaining. Images were captured from at least five randomly selected fields. Scale bar = 100 μm. Myotube mass indices (MMIs) were calculated as the area of MyHC‐positive myotube divided by the number of nuclei (only myotubes with more than seven nuclei were counted). (b) SUnSET assay analysis. Symbols (+) denote the presence of puromycin and/or CXCL14; additional + indicates higher concentration. Quantification of newly synthesized proteins was performed using the SUnSET assay by dividing puromycin intensity by total proteins. (c) Western blot analysis of MyHC isoforms in myotubes treated with various concentrations of CXCL14 for 48 h. (d) Western blot analysis of AKT‐S6K and FOXO pathways in myotubes treated with CXCL14 for 48 h at 100 ng/mL. (e) Immunofluorescence images of myotubes treated with siRNA (si‐NT or si‐*Rps6kb1* [S6K]), followed by CXCL14 treatment. Myotubes were stained for MyHC (red) and nuclei (blue) using an anti‐MyHC antibody and DAPI, respectively. Scale bar = 100 μm. MMI calculations are shown. (f) Western blot analysis of MyHC isoform expression in myotubes treated with siRNA in the presence or absence of CXCL14. Gene knockdown efficiency of each siRNA was verified by Western blot analysis. Data are presented as mean ± SEM The *p* values were calculated by one‐way ANOVA with Tukey's post hoc test. *p ≤ 0.05; **p ≤ 0.01; ***p ≤ 0.001; ****p ≤ 0.0001 Quantification of all Western blot data is available in Figure S2.

To investigate the role of CXCL14 in regulating skeletal muscle mass, we used C2C12 cell‐derived myotubes differentiated over 4 days. These myotubes were subsequently cultured in high‐serum growth medium to minimize further differentiation and cell fusion, treated with CXCL14 and immunostained for myosin heavy chain (MyHC) expression. The myotube mass index (MMI, in square micrometres) was calculated by dividing the area of MyHC‐positive myotubes containing more than five nuclei by the number of nuclei in the same myotubes. CXCL14 treatment significantly increased the MMI in a dose‐dependent manner (Figure 1a and Table S1), indicating that CXCL14 induces myotube hypertrophy.

While CXCL14 induced hypertrophy in differentiated myotubes, we also aimed to determine whether CXCL14 affects myogenic differentiation and cell fusion. C2C12 cells were differentiated in differentiation medium containing 2% horse serum, with or without CXCL14, for up to 4 days. Differentiation indices (the number of nuclei in the marker‐positive cells divided by the total number of nuclei) were assessed at 2 days by measuring Myogenin (MyoG) expression (Figure S1a and Table S2a), and at 4 days by MyHC immunostaining (Figure S1b and Table S2b). At both time points, differentiation indices of CXCL14‐treated cells were comparable to those of untreated control cells. Western blot analysis also showed no significant changes in the expression of myogenic differentiation markers (PAX7, Myf5, MyoD and MyoG) and MyHC isoforms by CXCL14 treatment (Figure S1c). Although CXCL14 did not impact myogenic differentiation, CXCL14 significantly reduced mononuclear myocytes while increasing myotubes with more than five nuclei, suggesting that CXCL14 may enhance cell fusion during differentiation (Figure S1b and Table S2c).

CXCL14‐induced myotube hypertrophy suggests a potential change in protein metabolism. To investigate the effects of CXCL14 on global protein synthesis, the surface sensing of translation (SUnSET) assay was employed. Puromycin, structurally similar to aminoacyl‐tRNA, can be incorporated into newly synthesized peptides, serving as a probe for assessing the rate of de novo protein synthesis. CXCL14 treatment for 30 min effectively increased puromycin incorporation in myotubes, confirming that CXCL14 enhances global protein synthesis in myotubes (Figure 1b). Given that MyHCs are the predominant proteins in myotubes, we evaluated the expression of total MyHC and three isoforms (Figures 1c and S2a). CXCL14 treatment increased all examined MyHC isoforms and total MyHC, reinforcing the link between CXCL14‐induced myotube hypertrophy and enhanced protein synthesis.

Next, we investigated how CXCL14 regulates protein metabolism signalling in myotubes. C2C12‐derived myotubes were treated with CXCL14 for either 48 h (Figure 1d) or subjected to a time course of up to 2 h (Figure S2c). After 48 h, CXCL14 treatment increased phosphorylation of AKT at Ser473 and Thr308 residues, indicating dose‐dependent AKT activation (Figures 1d and S2b). Consequently, phosphorylation of 4EBP1, S6K and FOXO—key targets of activated AKT—also increased (Figure 1d). Enhanced phosphorylation of S6 ribosomal protein, a target of activated S6K, was subsequently observed. Consistent with 48‐h treatment, similar AKT activation and downstream signalling were also observed during brief exposure to CXCL14, with peak AKT and S6K phosphorylation occurring within 15 to 30 min (Figure S2c). However, 4EBP1 phosphorylation was less pronounced during short CXCL14 treatment. Notably, FOXO1 and FOXO3 phosphorylation increased within 15 min of CXCL14 treatment, indicating that CXCL14 rapidly activates AKT and downstream signalling.

To further confirm that CXCL14‐induced hypertrophy is primarily mediated by increased protein synthesis, we knocked down the *Rps6kb1* gene, which encodes S6K and directly regulates protein synthesis (Figure 1e,f). C2C12‐derived myotubes were transfected with *Rps6kb1*‐specific small interfering RNA (siRNA), and effective gene knockdown was verified (Figure 1f). MMIs were calculated from MyHC immunostaining (Figure 1e and Table S4). In myotubes transfected with *Rps6kb1*‐specific siRNA, CXCL14 no longer induced hypertrophy, while it successfully promoted hypertrophy in nonspecific siRNA‐transfected myotubes. Additionally, CXCL14 failed to enhance MyHC expression in *Rps6kb1*‐knockdown myotubes (Figures 1f and S2d). These results corroborate that CXCL14 induces myotube hypertrophy via AKT‐S6K‐mediated protein synthesis.

### 3.2 Cxcl14 Overexpression in the TA Muscles of the Mouse Hindlimb Leads to Muscle Hypertrophy and Activation of the AKT Signalling Pathway

**FIGURE 2:**
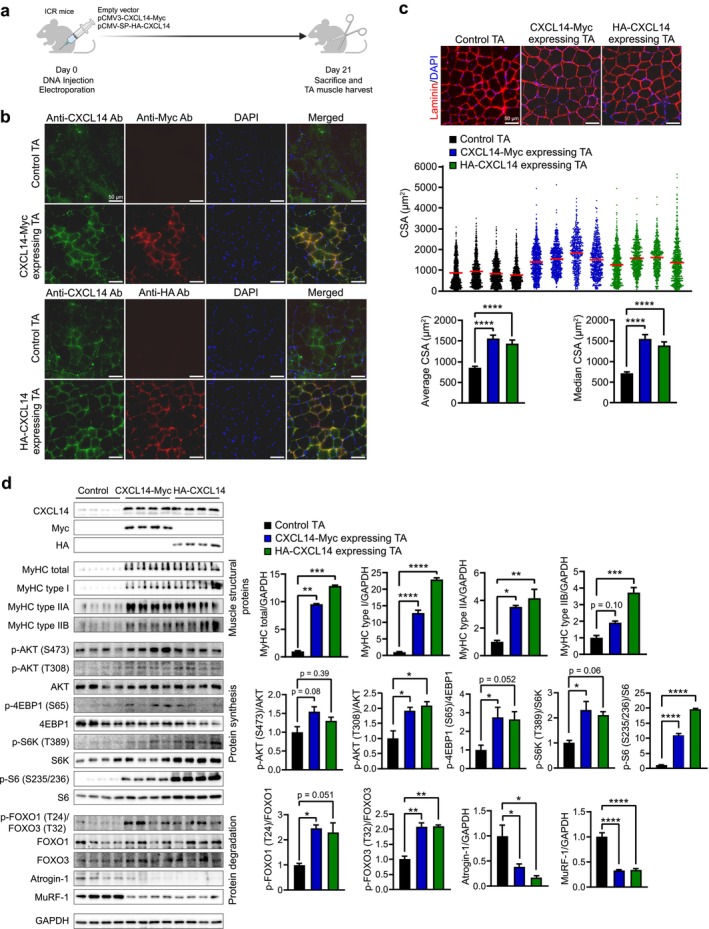
*Cxcl14* overexpression promotes TA muscle hypertrophy by activating the AKT‐S6K pathway and inhibiting the FOXO‐Atrogin‐1/MuRF‐1 pathway. (a) Experimental design for *Cxcl14* overexpression in TA muscles in mice. Control plasmid (*n* = 4) and plasmids expressing CXCL14‐Myc (*n* = 4) or HA‐CXCL14 (*n* = 4) were electroporated into the TA muscles of ICR mice, which were harvested after 21 days. (b) TA muscle sections were immunofluorescently stained with anti‐CXCL14 (green), anti‐Myc (red) or anti‐HA (red) antibodies to confirm *Cxcl14* overexpression. Sections were counterstained with DAPI for nuclei (blue). Scale bar = 50 μm. (c) TA muscle sections were immunofluorescently stained with an anti‐Laminin antibody (red) and counterstained with DAPI for nuclei (blue). All images were captured from at least five randomly selected fields. Distribution of CSA in control (black), CXCL14‐Myc (blue) and HA‐CXCL14 (green) TA muscles is shown in the middle, with average and median CSA presented at the bottom. To calculate *p* values, one‐way ANOVA with Tukey's post hoc test was used for average CSA, while Kruskal‐Wallis with Dunn's post hoc test was applied for median CSA. (d) Western blot analysis of TA muscle lysates. Endogenous and overexpressed CXCL14 were analysed in *Cxcl14*‐overexpressed TA muscles using anti‐CXCL14, anti‐Myc and anti‐HA antibodies. Glyceraldehyde 3‐phosphate dehydrogenase (GAPDH) expression was used for normalization. MyHC isoform expression and AKT‐S6K and FOXO pathway activity were assessed by specific antibodies as indicated. Quantification of Western blot data is shown on the right. Data are presented as mean ± SEM The *p* values were calculated by one‐way ANOVA with Tukey's post hoc test. *p ≤ 0.05; **p ≤ 0.01; ***p ≤ 0.001; ****p ≤ 0.0001.

To determine the hypertrophic activity of CXCL14 in vivo, *Cxcl14* expression plasmid or control plasmid DNA was electroporated into the TA muscles of 8‐week‐old ICR mice, as previously described [16]. Two *Cxcl14* expression plasmids were used: pCMV3‐SP‐HA‐CXCL14 (HA‐epitope tagged at the N terminus of CXCL14) and pCMV3‐CXCL14‐Myc (Myc‐epitope tagged at the C terminus of CXCL14) (Figure 2a). Three weeks post electroporation, TA muscles were harvested for immunofluorescence staining and Western blot analyses. Efficient *Cxcl14* expression was confirmed by immunofluorescence staining (Figure 2b) and Western blot analysis (Figure 2d) using anti‐CXCL14 and anti‐epitope specific antibodies. In control TA muscles, CXCL14 expression was primarily detected in the interstitial space, while electroporated TA muscles showed elevated CXCL14 expression in certain myofibers and surrounding interstitial space, overlapping with signals detected by anti‐Myc or anti‐HA antibodies.

The CSA of TA muscles was measured to evaluate whether *Cxcl14* overexpression induced muscle hypertrophy. An anti‐Laminin antibody was used to stain the periphery of each myofiber. Both HA‐CXCL14‐ and CXCL14‐Myc‐expressing TA muscles showed significant increases in CSA compared to control muscles (Figure 2c and Table S5). Myofibers with CSA < 1000 μm^2^ were significantly reduced in CXCL14‐expressing muscles, while those with CSA > 1500 μm^2^ were considerably increased. The median and average CSA of myofibers also rose in CXCL14‐overexpressing muscles compared to control muscles, indicating strong evidence of muscle hypertrophy induced by *Cxcl14* overexpression.

To determine if *Cxcl14* overexpression enhances protein synthesis in TA muscles, MyHC expression and AKT signalling cascade were analysed in TA muscles electroporated with *Cxcl14* expression plasmids (Figure 2d). Consistent with in vitro results (Figure 1c), notable increases in MyHC isoform protein expression (Figure 2d) were detected in *Cxcl14*‐expressing TA muscles. The effects of *Cxcl14* overexpression were not fibre type‐specific, as the fibre type proportion relative to total fibres remained unchanged (Figure S3a,b), while hypertrophy occurred across all fibre types (Figure S3c and Table S6).

*Cxcl14* overexpression induced the phosphorylation of AKT and its downstream mediators, 4EBP1 and S6K (Figure 2d). Simultaneously, FOXO transcription factors were phosphorylated by activated AKT, leading to the inactivation of FOXO1/3 and subsequent down‐regulation of their target gene products, Atrogin‐1 and MuRF‐1. These in vivo results confirm that TA muscle hypertrophy from *Cxcl14* overexpression resulted from the increased protein synthesis and the suppression of FOXO‐dependent protein degradation through the AKT signalling cascade.

### 3.3 Transcriptome Analysis of CXCL14‐Overexpressing Skeletal Muscle Indicates Active Regulation of Target Genes Associated With FOXO1 and Nuclear Factor‐κB (NF‐κB) Transcription Factors

To assess the effects of *Cxcl14* overexpression on global transcription in skeletal muscles, we performed RNA‐seq transcriptome analysis on total RNA samples isolated from TA muscles electroporated with *Cxcl14* expression (*n* = 3) and control plasmids (*n* = 3), respectively (Figure S4). We identified 1100 upregulated and 262 downregulated differentially expressed genes (DEGs) with > 1.5‐fold change, *p* < 0.05 and an FDR < 0.01. Pathway analysis using the ENRICHR online tool revealed that upregulated DEGs were significantly associated with cytokine signalling and immune responses, as indicated by Gene Ontology–Biological Processes (GO–BP), WikiPathways and Reactome analyses (Figure S5). These findings underscore the chemokine nature of CXCL14. Conversely, downregulated DEGs were linked to multiple gene transcription‐related terms in GO–BP, with associations to mitogen‐activated protein kinase (MAPK) signalling in WikiPathways and Reactome analysis.

**FIGURE 3:**
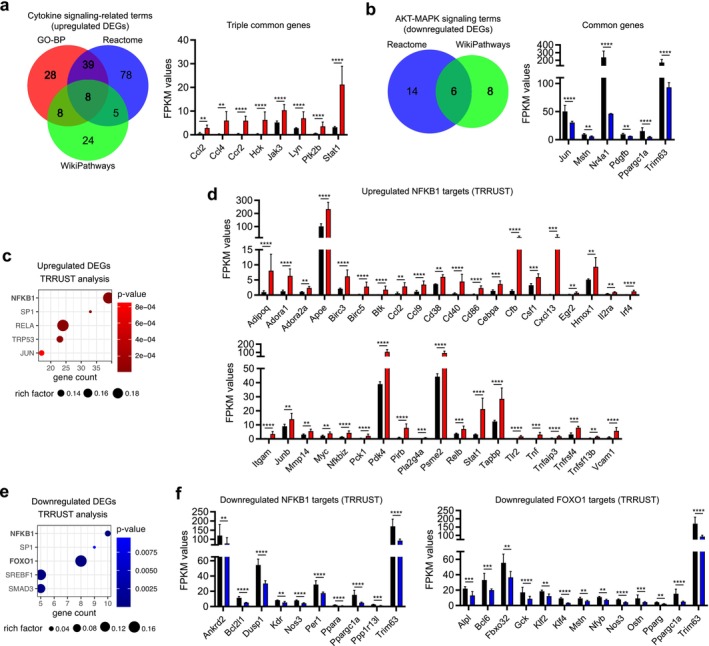
*Cxcl14* overexpression influences genes related to immune function, muscle mass regulation and FOXO pathway. (a) Venn diagram illustrating the upregulated DEGs associated with cytokine signalling‐related terms in GO–BP, Reactome and WikiPathways (left). The expression of DEGs commonly identified in all three annotation analyses is shown on the right. (b) Venn diagram depicting the downregulated DEGs associated with AKT–MAPK signalling‐related terms in Reactome and WikiPathways (left). The expression of DEGs commonly identified in both annotation analyses is shown on the right. (c) TRRUST analysis of upregulated DEGs, highlighting top five transcription factors associated with these genes. (d) Expression of upregulated DEGs linked to the NF‐κB transcription factor. (e) TRRUST analysis of downregulated DEGs, highlighting the top five transcription factors associated with these genes. (f) Expression of downregulated DEGs associated with FOXO and NF‐κB transcription factors. Data are presented as mean ± standard deviation. *p ≤ 0.05; **p ≤ 0.01; ***p ≤ 0.001; ****p ≤ 0.0001.

Cytokine signalling‐related terms and their associated genes were further examined (Figure 3a). Eight common genes were identified across all three annotation analyses, including chemokine genes, *Ccl2* and *Ccl4*, as well as the CCL2 receptor gene *Ccr2*. Downstream effector genes of chemokine signalling, *Stat1* and *Jak3*, were also identified. Interestingly, previous studies on CCL2/CCR2 signalling in muscle mass regulation have yielded conflicting results, showing both positive and negative roles [10, 11]. Additionally, *Cxcl14* overexpression increased the expression of two Src family kinase genes, *Hck* and *Lyn*, as well as the tyrosine kinase gene, *Ptk2b*, although their roles in skeletal muscle hypertrophy remain unclear.

For downregulated DEGs, we analysed the AKT‐MAPK signalling terms identified in Reactome and WikiPathways (Figure 3b). Six genes were common in both annotation analyses. The expression of two well‐known atrophic factor genes, *Mstn* (myostatin) and *Trim63* (MuRF‐1), was significantly downregulated by *Cxcl14* overexpression, supporting the anti‐atrophic activity of CXCL14. However, contrary to expectations, the *Nr4a1*, *Pdgfb* and *Ppargc1a* genes shown to induce muscle hypertrophy [17, 18, 19] were downregulated.

TRRUST (Transcriptional Regulatory Relationship Unraveled by Sentence‐based Text mining) analysis was conducted to elucidate the transcription factors regulating DEGs (Figure 3c,e). Thirty‐nine upregulated DEGs were significantly associated with NFKB1 and RELA transcription factors (Figure 3d). Conversely, 10 and 13 downregulated DEGs were linked to NFKB1 and FOXO1 transcription factors, respectively (Figure 3f). This suggests that NF‐κB factors may mediate CXCL14 signalling in both positive and negative regulation of DEGs, while FOXO1 appears to exclusively regulate DEGs negatively. Notably, *Ccl2* and *Stat1* among the upregulated DEGs are associated with NF‐κB [20, 21], while *Mstn* and *Trim63* among the downregulated DEGs are linked to FOXO1 [22, 23], with *Trim63* also a target of NFKB1 [24]. Overall, the transcriptomic analysis reveals that CXCL14 regulates genes involved in muscle mass control through NF‐κB and FOXO1 transcription factors.

### 3.4 CXCL14 Can Reverse Muscle Atrophy Induced by LPS Both In Vitro and In Vivo

Given the ability of CXCL14 to induce muscle hypertrophy both in vitro and in vivo, it is crucial to determine whether CXCL14 can reverse muscle atrophy caused by many pathological conditions. The muscle atrophy model induced by LPS was employed first. LPS is a major component of the bacterial cell membrane, and its treatment mimics bacterial infections [25].

**FIGURE 4:**
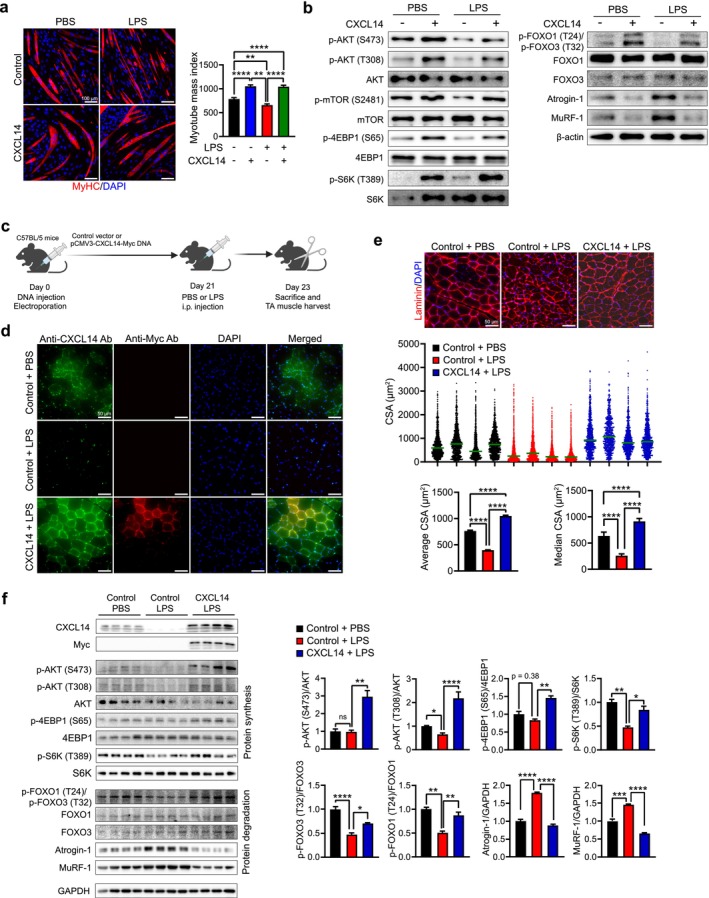
CXCL14 protects against LPS‐induced muscle atrophy in vitro and in vivo. (a) C2C12‐derived myotubes were treated with LPS, with or without CXCL14 (100 ng/mL). Myotubes were immunofluorescently stained with an anti‐MyHC antibody (red) and counterstained with DAPI (blue) for nuclei. Images were captured from at least five randomly selected fields. Scale bar = 100 μm. MMIs are presented as mean ± SEM (right). (b) Western blot analysis of AKT‐S6K and FOXO pathways in LPS‐treated myotubes, with or without CXCL14. *β*‐actin expression was used as a normalization control for both right and left panels. Quantification of Western blot data is presented in Figure S6a. (c) Experimental design for investigating *Cxcl14* overexpression in an LPS‐induced atrophy mouse model. C57BL/6N mice were electroporated with control (*n* = 8) or CXCL14‐Myc plasmid (*n* = 4) in the TA skeletal muscles. After 21 days, LPS or PBS was injected intraperitoneally into mice. Mice were sacrificed 2 days later. (d) TA muscle sections were immunofluorescently stained with anti‐CXCL14 (green) and anti‐Myc (red) antibodies, with DAPI (blue) for nuclei. Scale bar = 50 μm. (e) TA sections were immunofluorescently stained for Laminin expression (red) and counterstained with DAPI (blue) for nuclei. Images were captured from at least five randomly selected fields. Distribution of CSA in control (black), control + LPS (red) and CXCL14‐Myc + LPS (blue) is shown in the middle. The average and median CSA are presented as mean ± SEM (bottom). To calculate *p* values, one‐way ANOVA with Tukey's post hoc test was used for average CSA, while Kruskal–Wallis with Dunn's post hoc test was applied for median CSA. (f) Western blot analysis of TA muscle lysates. Both endogenous and overexpressed CXCL14 were analysed using anti‐CXCL14 and anti‐Myc antibodies, respectively. GAPDH expression was used for normalization. The activity of AKT‐S6K and FOXO pathways was assessed using specific antibodies as indicated. Quantification of Western blot data is presented on the right. Data are expressed as mean ± SEM. The *p* values were calculated by one‐way ANOVA with Tukey's post hoc test. *p ≤ 0.05; **p ≤ 0.01; ***p ≤ 0.001; ****p ≤ 0.0001.

C2C12‐derived myotubes were treated with LPS, with or without CXCL14. MyHC immunostaining was conducted to assess MMI (Figure 4a and Table S7). As anticipated, LPS treatment reduced MMI, indicating atrophy, compared to untreated controls. However, CXCL14 significantly reversed this atrophy, restoring MMI to control levels and altering the protein metabolism process (Figures 4b and S6a). The activity of the protein synthesis pathway suppressed by LPS, shown by decreased phosphorylation of AKT, mammalian target of rapamycin (mTOR), 4EBP1 and S6K, was restored to control levels by CXCL14. Additionally, LPS‐induced protein degradation, evidenced by increased expression of Atrogin‐1 and MuRF‐1, along with FOXO phosphorylation, was also reversed to control levels by CXCL14. Thus, CXCL14 effectively corrected the protein metabolism imbalance induced by LPS.

The potential of CXCL14 to alleviate muscle atrophy caused by LPS in vivo was also investigated. CXCL14‐Myc expression or control empty plasmid DNA was electroporated into the TA muscles of 8‐week‐old mice. After 3 weeks, LPS was injected intraperitoneally into the mice, and muscle atrophy was assessed 2 days later by measuring CSA of TA muscle (Figure 4c). As previously demonstrated [26], LPS administration in control mice resulted in significant TA muscle atrophy, characterized by an increased presence of smaller myofibers and reduction of both the mean and median CSA (Figure 4e). Interestingly, endogenous *Cxcl14* expression was markedly decreased in LPS‐administered mice compared to control mice (Figure 4d,f), indicating an inverse correlation between muscle atrophy and endogenous *Cxcl14* expression. When LPS was administered in *Cxcl14*‐overexpressing mice, TA muscles exhibited significant resistance to LPS‐induced muscle atrophy (Figure 4e and Table S8). The CSA distribution in *Cxcl14*‐overexpressing, LPS‐administered mice showed notable recovery from muscle atrophy, with both the mean and median CSA even surpassing those of control mice.

Next, the protein metabolism pathway was examined. In the LPS‐administered mice, the phosphorylation levels of AKT, 4EBP1 and S6K were lower than those in control mice, indicating reduced protein synthesis. Conversely, decreased phosphorylation of FOXO1/3 and increased expression of Atrogin‐1/MuRF‐1 demonstrated the activation of protein degradation in LPS‐administered mice (Figure 4f). However, *Cxcl14* overexpression in the TA muscles of LPS‐administered mice restored the balance in protein metabolism. The protein synthesis pathway was activated as evidenced by increased phosphorylation of AKT, 4EBP1 and S6K, while the protein degradation pathway was suppressed, characterized by increased phosphorylation of FOXO1 and decreased expression of Atrogin‐1/MuRF‐1.

### 3.5 CXCL14 Can Reverse Muscle Atrophy Induced by DEX Both In Vitro and In Vivo

To further demonstrate that CXCL14 can act as an anti‐atrophic factor under various atrophy conditions, we employed a second muscle atrophy model: a DEX‐induced one. While DEX is effective for treating several diseases, long‐term administration can lead to muscle atrophy [27].

**FIGURE 5:**
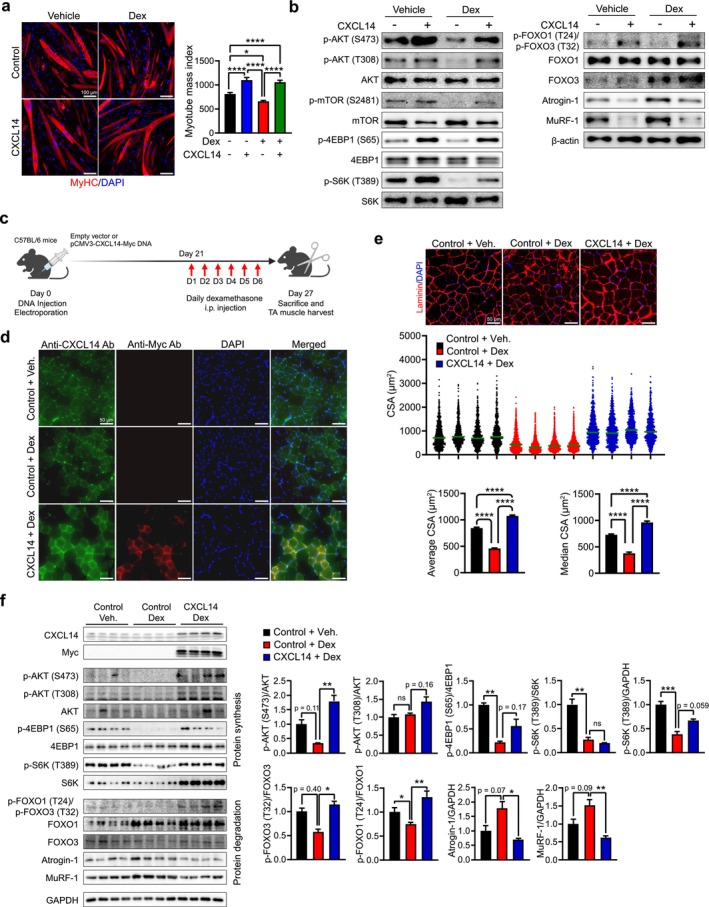
CXCL14 protects against DEX‐induced muscle atrophy. (a) C2C12‐derived myotubes were treated with DEX, with or without CXCL14 (100 ng/mL). Myotubes were immunofluorescently stained with an anti‐MyHC antibody (red) and counterstained with DAPI (blue) for nuclei. Images were captured from at least five randomly selected fields. Scale bar = 100 μm. MMIs are presented as mean ± SEM (right). (b) Western blot analysis of the AKT‐S6K and FOXO pathways in DEX‐treated myotubes with CXCL14. β‐actin expression was used as a normalization control for both right and left panels. Quantification of Western blot data is presented in Figure S6b. (c) Experimental design for investigating *Cxcl14* overexpression in a DEX‐induced atrophy mouse model. C57BL/6N mice were electroporated with control (*n* = 8) or CXCL14‐Myc plasmid (*n* = 4) in TA skeletal muscles. After 21 days, DEX or vehicle was injected intraperitoneally daily for 6 days. Mice were sacrificed the following day. (d) TA muscle sections were immunofluorescently stained with anti‐CXCL14 (green) and anti‐Myc (red) antibodies, with DAPI (blue) for nuclei. Scale bar = 50 μm. (e) TA sections were immunofluorescently stained for Laminin expression (red) and counterstained with DAPI (blue) for nuclei. Images were captured from at least five randomly selected fields. Distribution of CSA in control (black), control + DEX (red) and CXCL14‐Myc + DEX (blue) is shown in the middle panel. The average and median CSA are presented as mean ± SEM (bottom). To calculate *p* values, one‐way ANOVA with Tukey's post hoc test was used for average CSA, while Kruskal–Wallis with Dunn's post hoc test was applied for median CSA. (f) Western blot analysis of TA muscle lysates. Endogenous and overexpressed CXCL14 were analysed using anti‐CXCL14 and anti‐Myc antibodies, respectively. GAPDH expression was used for normalization. The activity of AKT‐S6K and FOXO pathways was assessed using specific antibodies as indicated. Quantification of Western blot data is shown on the right. Data are presented as mean ± SEM. The *p* values were calculated by one‐way ANOVA with Tukey's post hoc test. *p ≤ 0.05; **p ≤ 0.01; ***p ≤ 0.001; ****p ≤ 0.0001.

We investigated whether CXCL14 could protect muscle tissue from DEX‐induced atrophy. C2C12‐derived myotubes were treated with DEX, with or without CXCL14 (Figure 5a and Table S9). As previously shown [28], DEX treatment caused myotube atrophy, evidenced by a reduced MMI. However, co‐treatment with CXCL14 effectively reversed this atrophy, restoring MMI to levels similar to control myotubes. Additionally, CXCL14 restored the activity of protein metabolism pathways affected by DEX (Figures 5b and S6b). While DEX inhibited the AKT‐mTOR‐S6K pathway, CXCL14 alleviated this inhibition. Concurrently, the FOXO‐Atrogin‐1/MuRF‐1 pathway activated by DEX was suppressed by CXCL14. These findings indicate that CXCL14 can inhibit or protect against DEX‐induced atrophy in vitro.

To extend the in vitro findings to in vivo conditions, we performed electroporation‐mediated *Cxcl14* overexpression using pCMV3‐CXCL14‐Myc or a control empty plasmid DNA in the TA muscles of 8‐week‐old mice. Three weeks later, DEX was administered intraperitoneally daily for 6 days (Figure 5c). Unlike the LPS treatment, DEX did not reduce endogenous *Cxcl14* expression (Figure 5d,f). TA muscles electroporated with CXCL14 DNA exhibited elevated *Cxcl14* expression compared to control and DEX‐treated TA muscles (Figure 5d). As expected, DEX‐treated control mice displayed significant muscle atrophy, with a notable shift of CSA distribution pattern towards smaller CSA compared to control mice (Figure 5e and Table S10). However, *Cxcl14* overexpression in DEX‐treated TA muscles fully restored the CSA distribution to control levels, with both the mean and median CSA reflecting this recovery (Figure 5d,e and Table S10).

Furthermore, *Cxcl14* overexpression led to the activation of AKT‐S6K in DEX‐treated TA muscles, shown by increased phosphorylation of AKT, 4EBP1 and S6K (Figure 5f). In contrast, the activation of FOXO‐Atrogin‐1/MuRF‐1 pathway by DEX was reversed by *Cxcl14* overexpression. These results confirm that *Cxcl14* overexpression can counteract DEX‐induced atrophy by balancing protein metabolism, acting as an anti‐atrophic factor.

### 3.6 CXCL14 Can Induce Hypertrophy and Reverse Atrophy Caused by LPS and DEX in Human Skeletal Muscle Myotubes

**FIGURE 6:**
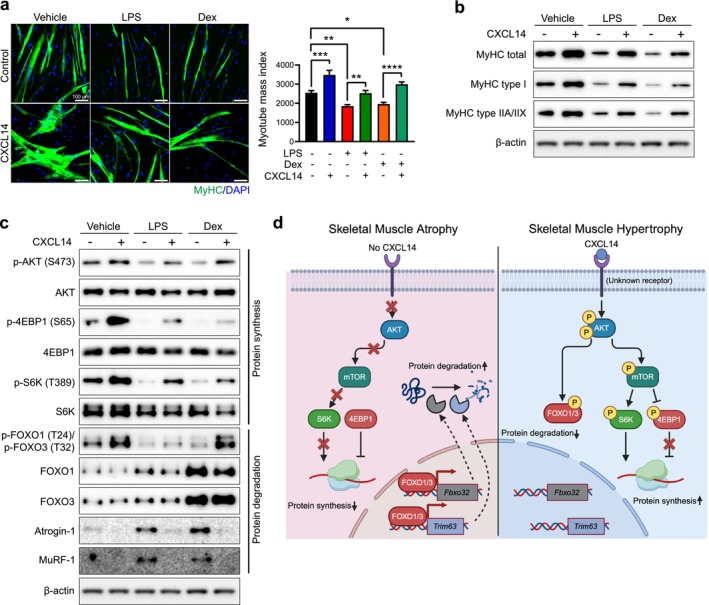
CXCL14 promotes hypertrophy and restored atrophy induced by LPS or DEX in differentiated primary human muscle myotubes. (a) Representative immunofluorescence images of differentiated human muscle cells treated with LPS or DEX, with or CXCL14 (100 ng/mL). Cells were immunofluorescently stained with an anti‐MyHC antibody (green) and DAPI (blue) for nuclei. Images were captured from at least six randomly selected fields. Scale bar = 100 μm. MMI calculations are shown. Data are presented as mean ± SEM The *p* values were calculated by one‐way ANOVA with Tukey's post hoc test. *p ≤ 0.05; **p ≤ 0.01; ***p ≤ 0.001; ****p ≤ 0.0001. (b and c) Western blot analysis of (b) MyHC isoform expression and (c) phosphorylation of AKT‐S6K and FOXO pathway components. Quantification of Western blot data is available in Figure S7. (d) Schematic model illustrating the function of CXCL14 in the regulation of skeletal muscle mass.

As a first step towards exploring the therapeutic potential of CXCL14 for skeletal muscle wasting or atrophy in humans, we determined whether CXCL14 positively regulates muscle mass through the AKT signalling cascade in human myotubes. Differentiated primary human myotubes were treated with human CXCL14 recombinant protein, with or without LPS or DEX. Similar to mouse myotubes, CXCL14 significantly increased the MMI in human myotubes compared to the control (Figure 6a and Table S11). Additionally, CXCL14 effectively reversed myotube atrophy induced by LPS or DEX, as indicated by the increase in MMI (Figure 6a and Table S11).

Next, we examined whether CXCL14 could enhance the expression of muscle structural proteins, specifically MyHC isoforms. CXCL14 increased the expression of both type I and type IIA/IIX MyHC isoforms, as well as total MyHC protein in primary human myotubes (Figures 6b and S7). The addition of LPS or DEX alone resulted in a significant reduction in MyHC expression; however, the presence of CXCL14 restored expression levels to those similar to the control group.

Finally, we explored the effect of CXCL14 on protein metabolism by assessing the AKT‐S6K and FOXO pathways. Consistent with the results obtained from both C2C12 myotubes in vitro and mouse TA muscle in vivo, CXCL14 reversed the inactivation of the AKT‐S6K pathway induced by LPS and DEX, while also reversing the activation of FOXO‐Atrogin‐1/MuRF‐1 pathway (Figures 6c and S7). Collectively, these results confirm that CXCL14 induces hypertrophy and exhibits an anti‐atrophy activity in human myotubes.

## 4 Discussion

### 4.1 CXCL14 Is a Novel Positive Regulator of Skeletal Muscle Mass via AKT‐S6K Signalling Pathway

Recent emerging research has highlighted the significant role of chemokines in regulating skeletal muscle mass [29, 30]. Given that FAP cell ablation in mice leads to profound muscle atrophy [13], CXCL14, a chemokine secreted predominantly by FAP cells in skeletal muscle [12], is a promising candidate that may play a crucial role in inducing hypertrophy and potentially preventing atrophy. Our findings provide direct evidence that CXCL14 functions as a novel hypertrophic factor of skeletal muscle, as both recombinant CXCL14 treatment in cultured myotubes and *Cxcl14* gene overexpression in TA muscles induced significant hypertrophy.

Previous studies indicate that CXCL14 activates AKT in non‐muscle cells, such as mammary fibroblasts [31] and human embryonic stem cells [32]. In skeletal muscle, CXCL14‐induced hypertrophy is closely associated with AKT activation via phosphorylation, as illustrated in the signalling model in Figure 6d. Active AKT phosphorylates both S6K (activating) and 4EBP1 (inactivating), leading to enhanced protein synthesis. Concurrently, AKT‐mediated phosphorylation of FOXO transcription factors results in their cytoplasmic sequestration, effectively downregulating Atrogin‐1 and MuRF‐1 key muscle‐specific ubiquitin E3 ligases that drive protein degradation.

Our study establishes that CXCL14 promotes muscle hypertrophy by shifting protein metabolism towards an anabolic state via the AKT‐S6K axis, while simultaneously suppressing catabolic signalling through FOXO inhibition. Notably, mice lacking the *Cxcl14* gene are significantly smaller than their wild‐type and heterozygous littermates [15], although the skeletal muscle phenotype in these animals remains unexplored. Future studies investigating the muscle phenotype of *Cxcl14‐null* mice will be crucial to elucidate its physiological role.

### 4.2 CXCL14 Exhibits Context‐Dependent Regulation of Myogenic Differentiation and Hypertrophy

CXCL14 plays multifaceted and context‐dependent roles in skeletal muscle. While our current study demonstrates that CXCL14 promotes myotube hypertrophy and muscle mass growth, earlier research has reported that it acts as a negative regulator of myogenesis in a cell‐autonomous manner in vitro [5]. Specifically, *Cxcl14* gene knockdown in C2C12 myoblasts enhances differentiation and cell fusion while reducing proliferation, effects that are reversed by recombinant CXCL14 treatment. In contrast, our findings show that exogenous CXCL14 has minimal impact on myogenic differentiation and only mildly increases cell fusion. This apparent discrepancy suggests that CXCL14's effects are dependent on the experimental context—particularly the mode of CXCL14 manipulation (genetic knockdown vs. protein treatment).

Furthermore, *Cxcl14* gene knockdown in injured mouse muscle has been shown to accelerate regeneration [5], implying a negative role for *Cxcl14* during muscle regeneration. The lentivirus‐mediated *Cxcl14* knockdown approach likely reduces *Cxcl14* expression not only in muscle cells but also in FAP cells in the injury area. Thus, *Cxcl14*, regardless of its source, may function as a negative regulator of muscle regeneration. To better delineate the specific roles of CXCL14 in muscle regeneration, we are currently developing a conditional knockout mouse model using tamoxifen‐inducible *Pax7‐CreERT* and *Pdgfrα‐CreERT* alleles to selectively delete *Cxcl14* in satellite cells and FAPs, respectively. These tools will allow us to dissect the cell‐type specific contributions of CXCL14 and clarify its function across distinct phases of muscle growth and regeneration.

### 4.3 CXCL14 Activate AKT Signalling via an Unidentified Receptor

A central question emerging from this study is: How does CXCL14 activate AKT signalling in skeletal muscle? CXCL14 is a promiscuous ligand known to interact with various receptors across different cell types. Previous studies have shown that CXCL14 can (1) allosterically bind to CXCR4, thereby inhibiting CXCL12 (SDF‐1) signalling in peripheral blood mononuclear cells [33]; (2) interact with the insulin‐like growth factor‐1 receptor (IGF‐1R) in human embryonic stem cells [32]; (3) signal through atypical chemokine receptor 2 (ACKR2) in breast [34] and non‐small‐cell lung [35] cancer cells; and (4) bind to G‐protein‐coupled receptor 85 (GPR85) in mammary fibroblasts [31]. Most recently, low‐density lipoprotein receptor‐related protein 1 (LRP1) has been identified as a putative receptor in immune cells [36]. Notably, LRP1 activation via Lactoferrin has been demonstrated to promote myotube hypertrophy in vitro [37], raising the possibility that LRP1 may also mediate CXCL14's anabolic effects in skeletal muscle.

Despite these promising leads, our RNA interference experiments targeting *Cxcr4*, *Igf‐1r* and *Lrp1* did not yield definite evidence implicating any of these receptors as functional mediators of CXCL14‐induced muscle growth (see Figure S8, Table S3). This suggests that either CXCL14 acts through a different, as‐yet‐unidentified receptor in muscle cells, or that its signalling involves a receptor complex or non‐canonical mechanism. The lack of a clearly defined receptor represents a major gap in our understanding of CXCL14's mode of action in muscle mass regulation. Future studies involving proteomic screening, CRISPR‐based knockout screening, or ligand‐receptor interactome mapping will be essential to fully elucidate the signalling mechanism and translate CXCL14 effects into potential therapeutic applications.

### 4.4 Could CXCL14 Serve as a Therapeutic Candidate for Muscle Atrophy?

To explore the therapeutic potential of CXCL14, we employed two well‐established muscle models of muscle atrophy. First, LPS‐induced atrophy represents an inflammation‐driven catabolic model, primarily mediated by activation of NF‐κB signalling, which upregulates protein degradation pathways [26]. Second, Dex‐induced atrophy mimics steroid myopathy, where AKT‐S6K signalling is suppressed, and protein degradation is enhanced via FOXO‐mediated transcription of atrogenes such as *Fbxo32* (Atrogin‐1) and *Trim63* (MuRF‐1) [27]. Our findings demonstrate that both recombinant CXCL14 treatment in vitro and *Cxcl14* gene overexpression in vivo effectively mitigate muscle atrophy in LPS‐ and DEX‐induced atrophy models. These results suggest that CXCL14 has broad therapeutic efficacy across distinct pathophysiological pathways that lead to muscle loss. This versatility underscores CXCL14's potential as a novel, endogenous anabolic factor for treating a wide range of muscle wasting disorders. To further validate its therapeutic applicability, future studies should investigate the systematic or intramuscular administration of recombinant CXCL14 protein in preclinical models of muscle atrophy, thereby confirming its bioactivity, safety and translational relevance.

## Conflicts of Interest

The authors declare no conflicts of interest.

## Supporting information


**Data S1:** Supplementary Information.

**Table S1:** Mass index of C2C12 myotubes treated with CXCL14 in growth medium.

**Table S2:** Effects of CXCL14 on myogenic differentiation in C2C12 cells.
**Table S3:** Mass index of C2C12 myotubes treated with CXCL14 in differentiation medium.
**Table S4:** Effect of Rps6kb1 gene knockdown on C2C12 myotube mass.
**Table S5:** Effect of Cxcl14 overexpression in cross‐sectional area (CSA) of TA muscle.
**Table S6:** Effect of Cxcl14 overexpression on cross‐sectional area (CSA) of different fibre types in TA muscle.
**Table S7:** Mass index of LPS‐treated C2C12 myotubes.
**Table S8:** Effect of Cxcl14 overexpression on TA muscle cross‐sectional area (CSA) in LPS‐treated mice.
**Table S9:** Mass index of dexamethasone‐treated C2C12 myotubes.
**Table S10:** Effect of Cxcl14 overexpression on TA muscle cross‐sectional area (CSA) in dexamethasone (Dex)‐treated mice TA muscle.
**Table S11:** Effect of CXCL14 on mass index of primary human myotubes treated with LPS or dexamethasone.
**Table S12:** Effect of putative CXCL14 receptor gene knockdowns on C2C12 myotube mass index.

**Figure S1:** jcsm70087‐sup‐0004‐Supplementary_Legends.docx. **No effect of CXCL14 on myogenic differentiation in vitro.** (a) C2C12 cells were differentiated for 2 days with or without recombinant CXCL14 protein (20 and 100 ng/mL). Myogenin (MyoG) protein expression was assessed by immunofluorescence staining with an anti‐MyoG antibody (green), while DAPI (blue) was employed for nuclear counterstaining. Scale bar = 100 μm. The differentiation index, defined as the percentage of MyoG‐positive nuclei over total nuclei, was calculated from more than 300 cells. (b) C2C12 cells were differentiated for 4 days with or without recombinant CXCL14 protein (20 and 100 ng/mL). MyHC protein expression was assessed by immunofluorescence staining with an anti‐MyHC antibody (red), with DAPI (blue) used for nuclear counterstaining. Scale bar = 100 μm. The differentiation index and fusion index (distribution of myocytes (mononuclear) and myotubes (2–4 nuclei and > 5 nuclei) in MyHC‐positive cells) were calculated from over 300 cells. (c) Western blot analysis of myogenic differentiation markers was performed, with CXCL14‐treated and control cells harvested at four time points (1, 2, 3, 4 days of differentiation). Quantification of the Western blot data is presented in the right panels. (d) C2C12 myotubes obtained after 4 days of differentiation treated with recombinant CXCL14 protein (20 and 100 ng/mL) for two days in differentiation medium. MyHC protein expression was assessed by immunofluorescence staining with an anti‐MyHC antibody (red), with DAPI (blue) used for nuclear counterstaining. Scale bar = 100 μm. Myotube mass indices (MMIs) were calculated as the area of MyHC‐positive myotube divided by the number of nuclei (only myotubes with more than seven nuclei were counted). (e) Western blot analysis of AKT and FOXO proteins expression in CXCL14‐treated and control myotubes. C2C12 myotubes were treated with CXCL14 for 2 days in differentiation medium. Quantification of the Western blot data is presented in the right panels. Data are expressed as mean ± SEM To calculate p‐values, one‐way ANOVA with Tukey's post hoc test was used for panels (a), (b), (d), and (e), while an unpaired student's t‐test was applied for panel (c). *p ≤ 0.05; **p ≤ 0.01; ***p ≤ 0.001.
**Figure S2:** jcsm70087‐sup‐0004‐Supplementary_Legends.docx. **Quantification of Western blot data following CXCL14 treatment.** (a‐b) Relative fold change in MyHC isoform expression (a), and phosphorylation of AKT‐S6K and FOXO1/3 pathway components (b) in myotubes treated with CXCL14 for 48 h. (c) Western blot analysis of AKT‐S6K and FOXO pathways in myotubes treated with CXCL14 for up to 2 h at 100 ng/mL (left). Relative fold change in phosphorylation of the AKT‐S6K (middle) and FOXO1/3 (right) pathway components over a time course of CXCL14 treatment. (d) Relative fold change in MyHC isoform expression in *Rps6kb1* knockdown C2C12‐derived myofibers treated with CXCL14. Data are presented as mean ± SEM To calculate p‐values, one‐way ANOVA with Tukey's post hoc test was used for panels (a), (b) and (d), while repeated measures ANOVA with Dunnett's post hoc test was applied for panel (c). *p ≤ 0.05; **p ≤ 0.01; ***p ≤ 0.001; ****p ≤ 0.0001.
**Figure S3: Cxcl14 overexpression does not affect myofiber type determination**. (a) TA muscle sections were immunofluorescently stained for the expression of Laminin (green) and various MyHC isoforms (red). All images were captured in at least five randomly selected fields. Scale bar = 50 μm. (b) Percentage of different myofiber types relative to the total number of myofibers. (c) Distribution of CSA of different myofiber type in control (black), CXCL14‐Myc (blue), and HA‐CXCL14 (green) overexpressed TA muscles are presented on the left. Average and median CSA are shown on the right. Data are expressed as mean ± SEM (C and D). To calculate p‐values, one‐way ANOVA with Tukey's post hoc test was used for average CSA, while Kruskal‐Wallis with Dunn's post hoc test was applied to median CSA. *p ≤ 0.05; **p ≤ 0.01; ***p ≤ 0.001; ****p ≤ 0.0001.
**Figure S4: Transcriptome analysis of Cxcl14‐overexpressed TA muscles.** (a) Workflow for transcriptome analysis. (b) Volcano plot of DEGs. Blue and red dots represent downregulated and upregulated DEGs, respectively.
**Figure S5: Annotation analyses with DEGs in Cxcl14‐overexpressed TA muscles.** (a) Top 15 GO‐BP terms enriched in upregulated (red) and downregulated (blue) DEGs. (b) Top 15 WikiPathways terms enriched in upregulated (red) and downregulated (blue) DEGs. (c) Top 15 Reactome terms enriched in upregulated (red) and downregulated (blue) DEGs.
**Figure S6: Quantification of Western blot data from LPS‐ or DEX‐induced atrophy restored by CXCL14 in C2C12‐derived myotubes.** Relative fold change in the expression of AKT‐S6K and FOXO1/3 pathway components in (a) LPS‐ and (b) DEX‐induced atrophic myotubes, with or without CXCL14. Data are presented as mean ± SEM. One‐way ANOVA with Tukey's post hoc test was used for p‐value calculation. *p ≤ 0.05; **p ≤ 0.01; ***p ≤ 0.001; ****p ≤ 0.0001.
**Figure S7: Quantification of Western blot data from LPS‐ and DEX‐treated primary human muscle cells.** Relative fold change in the expression of different (a) MyHC isoforms, along with components of the (b) AKT‐S6K and (c) FOXO1/3 pathways, in LPS‐ and DEX‐treated myotubes, with or without CXCL14. Data are expressed as mean ± SEM. One‐way ANOVA with Tukey's post hoc test was used to calculate p‐values. *p ≤ 0.05; **p ≤ 0.01; ***p ≤ 0.001; ****p ≤ 0.0001.
**Figure S8: CXCL14‐induced hypertrophy is independent of known CXCL14 receptors.** C2C12 cell‐derived myotubes were transfected with siRNAs [si‐NT (*NonTarget)*, si‐*Cxcr4*, si‐*Igf1r*, or si‐*Lrp1*] and treated with CXCL14. Myotubes were stained with anti‐MyHC antibody (red) and counterstained with DAPI (blue) for nuclei to determine MMI. Scale bar = 100 μm. Gene knockdown efficiency by siRNAs was determined by Western blot analysis. Data are presented as mean ± SEM. One‐way ANOVA with Tukey's post hoc test was used to calculate p‐values. *p ≤ 0.05; **p ≤ 0.01; ***p ≤ 0.001; ****p ≤ 0.0001.
