# Supplementary material for: CXCL14 Promotes Skeletal Muscle Mass Growth and Attenuates Lipopolysaccharide‐ and Dexamethasone‐Induced Muscle Atrophy in Cultured Myotubes and Mouse Models
Source: J Cachexia Sarcopenia Muscle. 2025 Oct 14;16(5):e70087. doi: 10.1002/jcsm.70087 (PMC12519514; doi:10.1002/jcsm.70087)
Supplement: Supplementary file 1 — Data S1: Supplementary Information. [file JCSM-16-e70087-s001.docx]

**Supplementary materials and methods**

**Cell culture**

The C2C12 mouse myoblast cell line was cultured in the growth medium composed of Dulbecco’s modified Eagle’s medium (DMEM; Corning, 10-013-CVRC) supplemented with 10% fetal bovine serum (Corning, 35-015-CV) and 1% penicillin–streptomycin (Corning, 30-002-CI) at 37°C in a humidified incubator with 5% CO_2_. To induce myogenic differentiation, confluent cells were switched to the differentiation medium, which consists of DMEM with 2% heat-inactivated horse serum (Gibco, 26050-088) and 1% penicillin-streptomycin. The differentiation medium was refreshed every 2 days. After 4 days, or as specified in the figure legends, C2C12 myotubes were returned to growth medium before being used in experiments. C2C12 myotubes responded to the CXCL14 treatment in both growth (high-serum) and differentiation (low-serum) medium (Figures 1a, d and S1d, e) in a similar manner. A high-serum medium was used during CXCL14 treatment to minimize further differentiation and cell fusion.

Human primary skeletal muscle myoblasts (hSkM) were maintained in the growth medium. Differentiation was induced by switching to differentiation medium, which consists of low glucose DMEM (Gibco, 1885-084) with 2% normal horse serum (Gibco, 16050-122) and 1% penicillin-streptomycin. Fully differentiated myotubes were formed after 6 days, and the differentiation medium was substituted with the growth medium prior to CXCL14 treatment.

Recombinant mouse CXCL14 (R&D Systems, 730-XC-025), recombinant human CXCL14 (R&D Systems, 866-CX-025), LPS (Sigma-Aldrich, L3129), and DEX (Sigma-Aldrich, D2915) were used at the concentrations and durations indicated in the figure legends.

***In vivo* electroporation**

Electroporation was performed using Aihara and Miyazaki protocol with minor modifications [1]. After longitudinal plasmid injection into the TA muscles, stainless-steel electrode needles (4 mm apart) were inserted along the TA muscle. Plasmid DNA encoding mouse *Cxcl14* gene with a c-Myc or HA tag (SinoBiological, MG50141-CM and MG50141-NY) or an empty vector were used. Eight square-wave pulses (80 V, 20 ms/pulse) were applied at a rate of 1 pulse/s using an ECM 830 Electroporation System (BTX, USA). Mice were then placed on a heating pad for recovery. TA muscles were harvested 3 weeks after electroporation.

For atrophy model experiments, mice were electroporated with with a *Cxcl14*-Myc plasmid. Three weeks later, mice received either a single intraperitoneal (i.p.) LPS injection (1 mg/kg BW) or daily DEX injections (20 mg/kg BW) for 6 days. TA muscles were harvested 2 days after LPS injection or 1 day after the final DEX injection. TA muscles were snap-frozen in liquid nitrogen and stored at -80°C for further analysis.

**siRNA transfection**

ON-TARGETplus siRNAs targeting mouse *Cxcr4*, *Igf1r*, *Lrp1*, and *Rps6kb1*, along with control nontarget siRNA, were obtained from Horizon Discovery (Waterbeach, UK). siRNA transfection was performed using Lipofectamine™ RNAiMAX (Invitrogen, 13778100) following a reverse transfection protocol. siRNAs were diluted in serum-free Opti-MEM™ medium, mixed with Lipofectamine at a 1:3 ratio, and added to plates containing newly seeded C2C12 myoblasts. After overnight incubation at 37°C in a humidified incubator with 5% CO_2_, the growth medium was added, and myogenic differentiation was induced 2 days later.

**SUnSET assay**

The SUnSET assay was performed as previously described to assess protein synthesis [2]. Briefly, C2C12 myotubes were serum-starved for 1 hour and treated with 1 µM puromycin (Gibco, A11138-03), with or without CXCL14 (20 ng/mL or 100 ng/mL) in growth medium for 30 minutes. Cells were then harvested and analyzed by Western blot using an anti-puromycin antibody (Merck, MABE343).

**Western blot analysis**

Cells were lysed in Laemmli buffer on ice for 30 minutes, followed by protein denaturation at 95°C for 5 minutes. TA muscles were lysed in RIPA buffer with protease and phosphatase inhibitors under the same conditions. Protein concentrations for *in vivo* samples were determined using a SMART™ BCA Protein Assay Kit (Intron Biotechnology, 21071). Protein samples (20 µg) were separated by sodium dodecyl sulfate–polyacrylamide gel electrophoresis (6%, 8%, or 10%) and transferred to polyvinylidene difluoride membranes (Cytiva, 10600021). After blocking with 5% skim milk or bovine serum albumin (BSA) in TBS-Tween 20, the membranes were incubated with primary and subsequently HRP-conjugated secondary antibodies. After HRP reaction was performed using ECL Western blotting detection kit (GE Healthcare), visualization was conducted using the Amersham Imager 600 (GE Healthcare) at the Soonchunhyang Biomedical Science Core Facility.

The primary antibodies used in this study included anti-PAX7 (DSHB), anti-MyoG (Santa Cruz, sc-52903), anti-MYOD (Santa Cruz, sc-32758), anti-MYF5 (Santa Cruz, sc-302), anti-phospho-AKT (Ser^473^) (CST, 4060), anti-phospho-AKT (Thr^308^) (CST, 13038), anti-AKT (CST, 9272), anti-phospho-mTOR (Ser^2481^) (CST, 2974), anti-mTOR (CST, 2983), anti-phospho-4EBP1 (Ser^65^) (CST, 9451), anti-4EBP1 (CST, 9644), anti-phospho-S6K (Thr^389^) (CST, 9234), anti-S6K (CST, 2708), anti-phospho-S6 ribosomal protein (Ser^235/236^) (CST, 2211), anti-S6 ribosomal protein (CST, 2217), anti-CXCR4 (Abcam, ab181020), anti-IGF-1R (CST, 9750), anti-phospho-FOXO1 (Thr^24^)/FOXO3 (Thr^32^) (CST, 9464), anti-FOXO1 (CST, 2880), anti-FOXO3 (CST, 2497), anti-Atrogin-1 (Santa Cruz, sc-166806), anti-MuRF-1 (Santa Cruz, sc-398608), anti-β-actin (Sigma, A5441), anti-GAPDH (CST, 5174), anti-sarcomere MyHC (deposited to DSHB by Fischman, D.A., MF-20), anti-MyHC type I (deposited to DSHB by Schiaffino, S., BA-D5), type IIA (deposited to DSHB by Schiaffino, S., SC-71), and type IIB (deposited to DSHB by Schiaffino, S., BF-F3) antibodies.

**Histology and immunofluorescence staining**

Cells in culture were fixed with 3.7% formaldehyde in phosphate-buffered saline (PBS) for 15 minutes, washed with PBS, permeabilized with 1% IGEPAL in PBS, and blocked with 5% serum in 3% BSA in PBS prior to primary and secondary antibody incubation. DAPI (1:1000, Invitrogen, D3571) was used to stain the nuclei.

TA muscle tissues were fixed in 4% paraformaldehyde in PBS for up to 20 hours, washed in distilled water, processed in alcohol series followed by Xylene, paraffin-embedded, and sectioned at a thickness of 7 µm. The slides were deparaffinized, rehydrated, and underwent antigen retrieval, followed by blocking with 5% serum in PBS containing 0.2% Triton X-100. The slides were then incubated with primary and secondary antibodies and mounted with Fluoroshield™ with DAPI (Sigma, F6057) or Vectashield (Vector Laboratories, H-1000-10).

The primary antibodies used included anti-MyoG (Santa Cruz, sc-52903), anti-sarcomere MyHC (DSHB, MF-20), anti-MyHC type I (DSHB, BA-D5), type IIA (DSH, SC-71), and type IIB (DSHB, BF-F3), anti-Laminin (Sigma, L9393), anti-CXCL14 (Invitrogen, PA5-106402), anti-c-Myc (Sigma, C3956), and anti-HA (Sigma, H6908) antibodies. The secondary antibodies including Cy3 donkey anti-mouse (Jackson ImmunoResearch, 715-165-150), Alexa Fluor® 488-donkey anti-mouse (Jackson ImmunoResearch, 715-545-150), Cy3 donkey anti-rabbit (Jackson ImmunoResearch, 711-165-152), and Alexa Fluor® 488-donkey anti-rabbit (Jackson ImmunoResearch, 711-545-152) antibodies were used.

**RNA isolation, RNA sequencing (RNA-seq) and data analysis**

Total RNA was isolated from C2C12 myotubes or TA muscles using TRIzol® reagent (Ambion, 15596018) according to the manufacturer’s instructions. RNA-seq, differentially expressed gene (DEG) list generation, and statistical analysis were conducted by DNA Link Inc. (Seoul, South Korea). DEGs were selected based on a 1.5-fold change, p <0.05, and false discovery rate (FDR) <0.01. Enrichment analysis for Gene Ontology–Biological Processes (GO-BP), Reactome, WikiPathways, and TRRUST was performed using the ENRICHR online tool (<https://maayanlab.cloud/Enrichr/>). The R packages (ggplot2, EnhancedVolcano) in R Studio and GraphPad Prism version 9.0 were used for figure generation.

**References**

1. Aihara H, Miyazaki J-i. Gene transfer into muscle by electroporation in vivo. Nature Biotechnology. 1998;16:867-70. doi:10.1038/nbt0998-867

2. Goodman CA, Mabrey DM, Frey JW, Miu MH, Schmidt EK, Pierre P, et al. Novel insights into the regulation of skeletal muscle protein synthesis as revealed by a new nonradioactive in vivo technique. FASEB J. 2011;25:1028-39. doi:10.1096/fj.10-168799
