# Supplementary material for: CXCL14 Promotes Skeletal Muscle Mass Growth and Attenuates Lipopolysaccharide‐ and Dexamethasone‐Induced Muscle Atrophy in Cultured Myotubes and Mouse Models
Source: J Cachexia Sarcopenia Muscle. 2025 Oct 14;16(5):e70087. doi: 10.1002/jcsm.70087 (PMC12519514; doi:10.1002/jcsm.70087)
Supplement: Supplementary file 2 — Table S1: Mass index of C2C12 myotubes treated with CXCL14 in growth medium. [file JCSM-16-e70087-s002.docx]

**Supplementary Tables**

**Table S1. Mass index of C2C12 myotubes treated with CXCL14 in growth medium**

| Experimental groups | Mean ± SEM (µm^2^) | 95% CI limits | | P-value  (vs. Control) |
| --- | --- | --- | --- | --- |
|  |  | Lower | Upper |  |
| Control | 897.9 ± 33.33 | 829 | 996 | - |
| 20 ng/mL CXCL14 | 1138 ± 51.4 | 1031 | 1245 | ≤ 0.001 |
| 100 ng/mL CXCL14 | 1345 ± 50.97 | 1237 | 1453 | ≤ 0.0001 |

**Table S2. Effects of CXCL14 on myogenic differentiation in C2C12 cells**

| 1. ***Differentiation index at differentiation day 2 by MyoG expression*** | | | | | |
| --- | --- | --- | --- | --- | --- |
| Experimental groups | Mean ± SEM (%) | 95% CI limits | | P-value (vs. Control) |  |
|  |  | Lower | Upper |  |  |
| Control | 31.38 ± 0.8032 | 29.56 | 33.19 | - |  |
| 20 ng/mL CXCL14 | 30.64 ± 1.475 | 27.3 | 33.98 | ns |  |
| 100 ng/mL CXCL14 | 29.03 ± 1.367 | 25.93 | 32.12 | ns |  |
|  |  |  |  |  |  |
| 1. ***Differentiation index at differentiation day 4 by MyHC expression*** | | | | | |
| Experimental groups | Mean ± SEM (%) | 95% CI limits | | P-value (vs. Control) |  |
|  |  | Lower | Lower |  |  |
| Control | 37.43 ± 1.211 | 34.69 | 40.17 | - |  |
| 20 ng/mL CXCL14 | 41.03 ± 0.6673 | 39.52 | 42.54 | ns |  |
| 100 ng/mL CXCL14 | 37.34 ± 1.496 | 33.95 | 40.72 | ns |  |
|  |  |  |  |  |  |
| 1. ***Fusion index at differentiation day 4*** | | | | | |
| Experimental groups | Mean ± SEM (%) | 95% CI limits | | P-value (vs. Control) |  |
|  |  | Lower | Lower |  |  |
| Control |  |  |  |  |  |
| - Mononuclear | 58.59 ± 2.234 | 52.39 | 64.79 | - |  |
| - 2-4 nuclei | 29.04 ± 2.047 | 23.36 | 34.72 | - |  |
| - ≥ 5 nuclei | 12.37 ± 0.7946 | 10.16 | 14.58 | - |  |
| 20 ng/mL CXCL14 |  |  |  |  |  |
| - Mononuclear | 49.63 ± 3.28 | 40.53 | 58.74 | 0.117 |  |
| - 2-4 nuclei | 30.13 ± 3.276 | 21.03 | 39.23 | ns |  |
| - ≥ 5 nuclei | 20.24 ± 1.202 | 16.9 | 23.58 | ≤ 0.01 |  |
| 100 ng/mL CXCL14 |  |  |  |  |  |
| - Mononuclear | 42.38 ± 3.136 | 33.68 | 51.09 | ≤ 0.001 |  |
| - 2-4 nuclei | 32.95 ± 2.099 | 27.12 | 38.78 | ns |  |
| - ≥ 5 nuclei | 24.67 ± 1.493 | 20.52 | 28.81 | ≤ 0.0001 |  |

**Table S3. Mass index of C2C12 myotubes treated with CXCL14 in differentiation medium**

| Experimental groups | Mean ± SEM (µm^2^) | 95% CI limits | | P-value (vs. Control) |
| --- | --- | --- | --- | --- |
|  |  | Lower | Upper |  |
| Control | 922.4 ± 41.38 | 837.7 | 1007 | - |
| 20 ng/mL CXCL14 | 985.3 ± 33.48 | 916.9 | 1054 | ns |
| 100 ng/mL CXCL14 | 1149 ± 45.4 | 1056 | 1242 | ≤ 0.001 |

**Table S4. Effect of *Rps6kb1* gene knockdown on C2C12 myotube mass**

| Experimental groups | Mean ± SEM (µm^2^) | 95% CI limits | | P-value |
| --- | --- | --- | --- | --- |
|  |  | Lower | Upper |  |
| *si-NT* | 778.1 ± 9.351 | 737.9 | 818.4 | - |
| *si-NT +* 100 ng/mL CXCL14 | 1067 ± 35.61 | 913.3 | 1220 | ≤ 0.0001 (vs. *si-NT*) |
| *si-Rps6kb1* | 575 ± 3.324 | 560.7 | 589.3 | ≤ 0.001 (vs. *si-NT*) |
| *si-Rps6kb1 +* 100 ng/mL CXCL14 | 559.7 ± 8.819 | 521.7 | 597.6 | ns (vs. *si-Rps6kb1*),  ≤ 0.0001 (vs. *si-NT*+CXCL14) |

**Table S5. Effect of *Cxcl14* overexpression in cross-sectional area (CSA) of TA muscle**

| Experimental groups | Mean ± SEM (µm^2^) | 95% CI limits | | P-value  (vs. Control) |
| --- | --- | --- | --- | --- |
|  |  | Lower | Upper |  |
| Control TA | 870.1 ± 11.25 | 848.1 | 892.2 | - |
| CXCL14-Myc expressing TA | 1499 ± 17.8 | 1464 | 1534 | ≤ 0.0001 |
| HA-CXCL14 expressing TA | 1408 ± 15.42 | 1378 | 1438 | ≤ 0.0001 |

**Table S6. Effect of *Cxcl14* overexpression on cross-sectional area (CSA) of different fiber types in TA muscle**

| 1. ***Myosin heavy chain type I fibers*** | | | | |
| --- | --- | --- | --- | --- |
| Experimental groups | Mean ± SEM (µm^2^) | 95% CI limits | | P-value  (vs. Control) |
|  |  | Lower | Upper |  |
| Control TA | 576.0 ± 16.59 | 542.5 | 609.5 | - |
| CXCL14-Myc expressing TA | 1330 ± 45.76 | 1236 | 1423 | ≤ 0.0001 |
| HA-CXCL14 expressing TA | 1202 ± 39.37 | 1122 | 1281 | ≤ 0.0001 |
|  |  |  |  |  |
| 1. ***Myosin heavy chain type IIA fibers*** | | | | |
| Experimental groups | Mean ± SEM (µm^2^) | 95% CI limits | | P-value  (vs. Control) |
|  |  | Lower | Upper |  |
| Control TA | 657.5 ± 13.09 | 631.7 | 683.3 | - |
| CXCL14-Myc expressing TA | 1503 ± 29.52 | 1445 | 1562 | ≤ 0.0001 |
| HA-CXCL14 expressing TA | 1578 ± 22.94 | 1533 | 1623 | ≤ 0.0001 |
|  |  |  |  |  |
| 1. ***Myosin heavy chain type IIB fibers*** | | | | |
| Experimental groups | Mean ± SEM (µm^2^) | 95% CI limits | | P-value  (vs. Control) |
|  |  | Lower | Upper |  |
| Control TA | 616.2 ± 6.979 | 602.5 | 629.9 | - |
| CXCL14-Myc expressing TA | 1446 ± 20.04 | 1407 | 1486 | ≤ 0.0001 |
| HA-CXCL14 expressing TA | 1434 ± 16.45 | 1401 | 1466 | ≤ 0.0001 |

**Table S7. Mass index of LPS-treated C2C12 myotubes**

| Experimental groups | Mean ± SEM (µm^2^) | 95% CI limits | | P-value |
| --- | --- | --- | --- | --- |
|  |  | Lower | Upper |  |
| PBS | 785.9 ± 28.88 | 727.3 | 844.5 | - |
| PBS *+* 100 ng/mL CXCL14 | 1052 ± 28.52 | 993.1 | 1110 | ≤ 0.0001 (vs. PBS) |
| LPS | 657.1 ± 24.67 | 607 | 707.2 | ≤ 0.01 (vs. PBS) |
| LPS *+* 100 ng/mL CXCL14 | 1044 ± 30.25 | 981.8 | 1105 | ≤ 0.0001 (vs. LPS) |

**Table S8. Effect of *Cxcl14* overexpression on TA muscle cross-sectional area (CSA) in LPS-treated mice**

| Experimental groups | Mean ± SEM (µm^2^) | 95% CI limits | | P-value  (vs. Control) |
| --- | --- | --- | --- | --- |
|  |  | Lower | Upper |  |
| Control + PBS | 763.8 ± 9.858 | 744.5 | 783.2 | - |
| Control + LPS | 376.4 ± 4.472 | 367.6 | 385.1 | ≤ 0.0001 |
| CXCL14 + LPS | 1025 ± 11.14 | 1004 | 1047 | ≤ 0.0001 |

**Table S9. Mass index of dexamethasone-treated C2C12 myotubes**

| Experimental groups | Mean ± SEM (µm^2^) | 95% CI limits | | P-value |
| --- | --- | --- | --- | --- |
|  |  | Lower | Upper |  |
| Vehicle | 811.8 ± 30.21 | 751.3 | 872.6 | - |
| Vehicle *+* 100 ng/mL CXCL14 | 1100 ± 55.1 | 986.6 | 1213 | ≤ 0.0001 (vs. Vehicle) |
| Dex | 659.1 ± 19.0 | 619.9 | 698.3 | ≤ 0.05 (vs. Vehicle) |
| Dex *+* 100 ng/mL CXCL14 | 1055 ± 41.12 | 971.5 | 1139 | ≤ 0.0001 (vs. Dex) |

**Table S10. Effect of *Cxcl14* overexpression on TA muscle cross-sectional area (CSA) in dexamethasone (Dex)-**

**treated mice TA muscle**

| Experimental groups | Mean ± SEM (µm^2^) | 95% CI limits | | P-value  (vs. Control) |
| --- | --- | --- | --- | --- |
|  |  | Lower | Upper |  |
| Control + Vehicle | 835.8 ± 8.605 | 818.9 | 852.7 | - |
| Control + Dex | 457.6 ± 4.365 | 449.1 | 466.2 | ≤ 0.0001 |
| CXCL14 + Dex | 1056 ± 10.82 | 1035 | 1078 | ≤ 0.0001 |

**Table S11. Effect of CXCL14 on mass index of primary human myotubes treated with LPS or dexamethasone**

| Experimental groups | Mean ± SEM (µm^2^) | 95% CI limits | | P-value |
| --- | --- | --- | --- | --- |
|  |  | Lower | Upper |  |
| PBS | 2549 ± 114.7 | 2310 | 2787 | - |
| PBS *+* 100 ng/mL CXCL14 | 3481 ± 242.6 | 2973 | 3989 | ≤ 0.001 (vs. PBS) |
| LPS | 1856 ± 76.54 | 1696 | 2015 | ≤ 0.01 (vs. PBS) |
| LPS *+* 100 ng/mL CXCL14 | 2530 ± 143.5 | 2234 | 2825 | ≤ 0.01 (vs. LPS) |
| Dex | 1959 ± 90.44 | 1771 | 2148 | ≤ 0.05 (vs. PBS) |
| Dex *+* 100 ng/mL CXCL14 | 2995 ± 122.9 | 2741 | 3249 | ≤ 0.0001 (vs. Dex) |

**Table S12. Effect of putative CXCL14 receptor gene knockdowns on C2C12 myotube mass index**

| 1. ***Cxcr4 gene knockdown*** |
| --- |

| Experimental groups | Mean ± SEM (µm^2^) | 95% CI limits | | P-value |
| --- | --- | --- | --- | --- |
|  |  | Lower | Upper |  |
| *si-NT* | 509.9 ± 21.06 | 462.3 | 557.6 | - |
| *si-NT+*100 ng/mL CXCL14 | 660.3 ± 27.54 | 600.8 | 719.8 | ≤ 0.01 (vs. *si-NT*) |
| *si-Cxcr4* | 483.6 ± 25.95 | 428.9 | 538.4 | - |
| *si-Cxcr4+*100 ng/mL CXCL14 | 604.5 ± 18.61 | 563.5 | 645.4 | ≤ 0.01 (vs. *si-Cxcr4*),  ns (vs. *si-NT*+CXCL14) |
|  |  |  |  |  |

| 1. ***Igf1r gene knockdown*** |
| --- |

| Experimental groups | Mean ± SEM (µm^2^) | 95% CI limits | | P-value |
| --- | --- | --- | --- | --- |
|  |  | Lower | Lower |  |
| *si-NT* | 838.8 ± 49.94 | 735.7 | 941.8 | - |
| *si-NT+*100 ng/mL CXCL14 | 1217 ± 61.86 | 1086 | 1347 | ≤ 0.0001 (vs. *si-NT*) |
| *si-Igf1r* | 619.1 ± 39.87 | 536.2 | 702 | - |
| *si-Igf1r+*100 ng/mL CXCL14 | 1101 ± 56.12 | 984.6 | 1218 | ≤ 0.0001 (vs. *si-Igf1r*),  ns (vs. *si-NT*+CXCL14) |
|  |  |  |  |  |

| 1. ***Lrp1 gene knockdown*** |
| --- |

| Experimental groups | Mean ± SEM (µm^2^) | 95% CI limits | | P-value |
| --- | --- | --- | --- | --- |
|  |  | Lower | Lower |  |
| *si-NT* | 464 ± 13.69 | 436.4 | 491.6 | - |
| *si-NT+*100 ng/mL CXCL14 | 793.6 ± 20.04 | 752.3 | 834.8 | ≤ 0.0001 (vs. *si-NT*) |
| *si-Lrp1* | 462.7 ± 11.09 | 440.1 | 485.2 | - |
| *si-Lrp1* 100 ng/mL CXCL14 | 771.6 ± 18.4 | 733.7 | 809.5 | ≤ 0.0001 (vs. *si-Lrp1*),  ns (vs. *si-NT*+CXCL14) |
