# Supplementary figures and images for: CXCL14 Promotes Skeletal Muscle Mass Growth and Attenuates Lipopolysaccharide‐ and Dexamethasone‐Induced Muscle Atrophy in Cultured Myotubes and Mouse Models
Source: J Cachexia Sarcopenia Muscle. 2025 Oct 14;16(5):e70087. doi: 10.1002/jcsm.70087 (PMC12519514; doi:10.1002/jcsm.70087)

Fig. S1

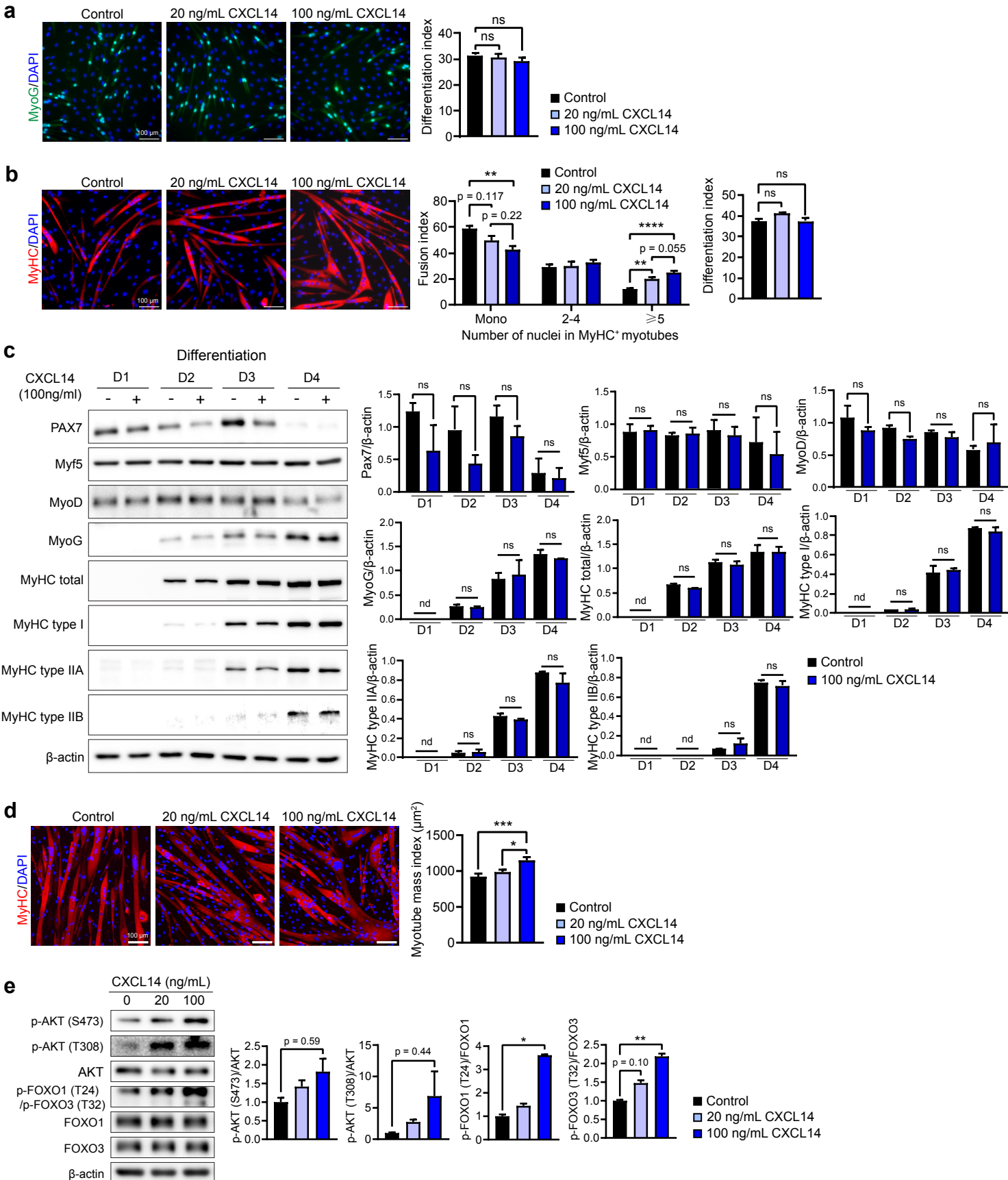

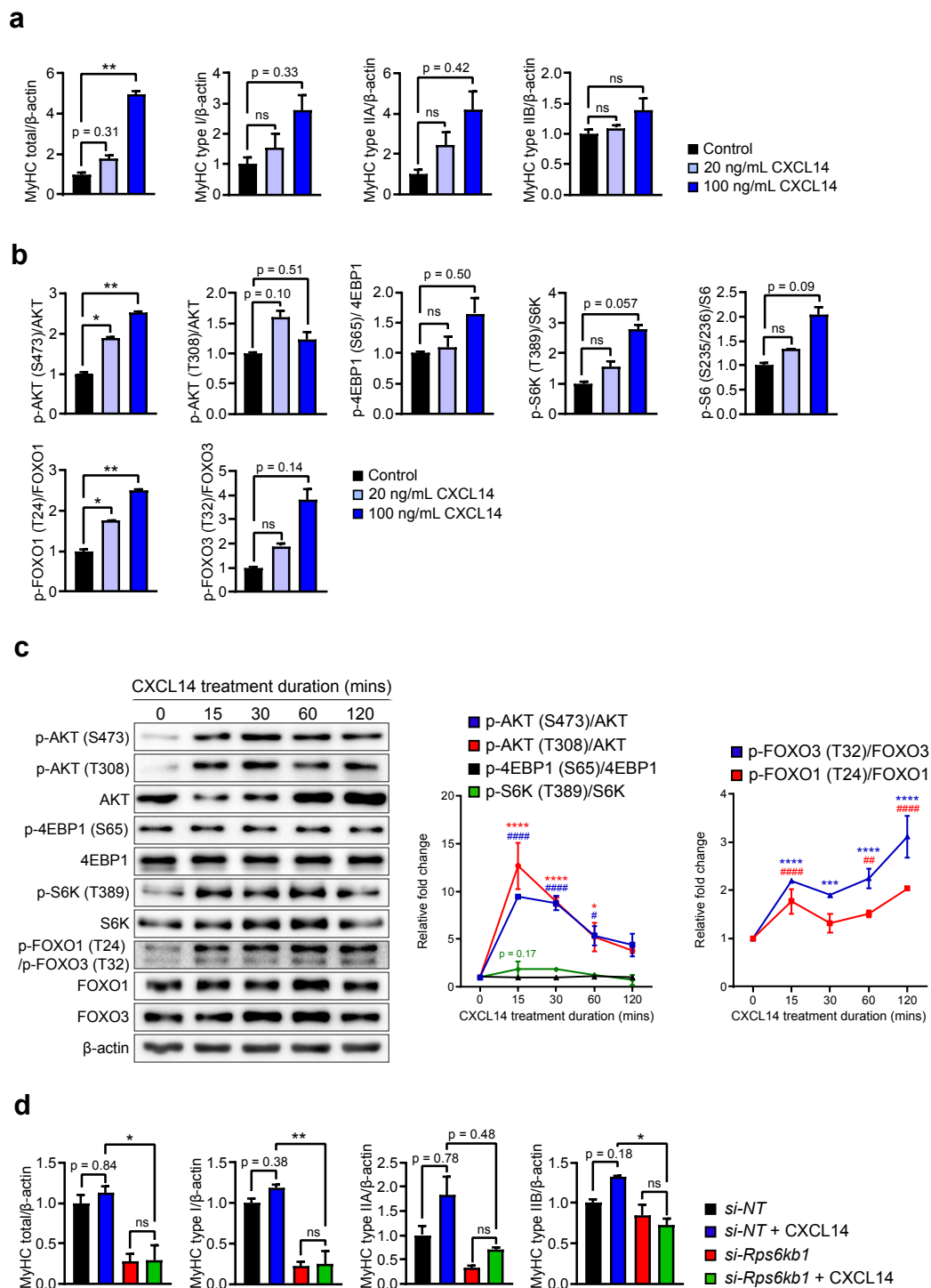

Fig. S3

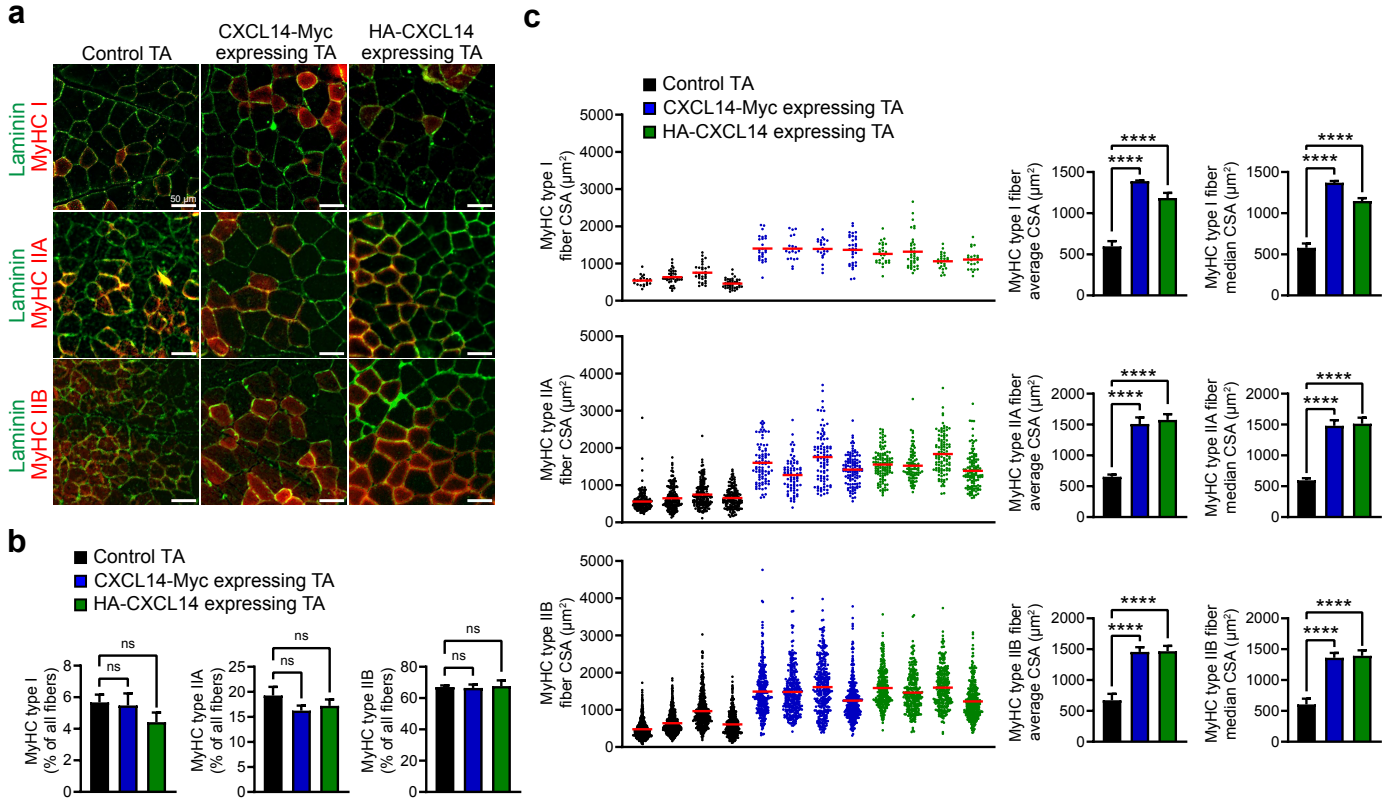

**a**

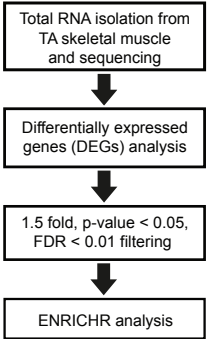

**b**

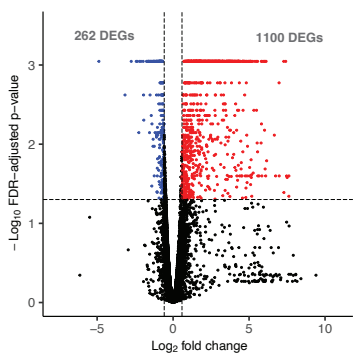

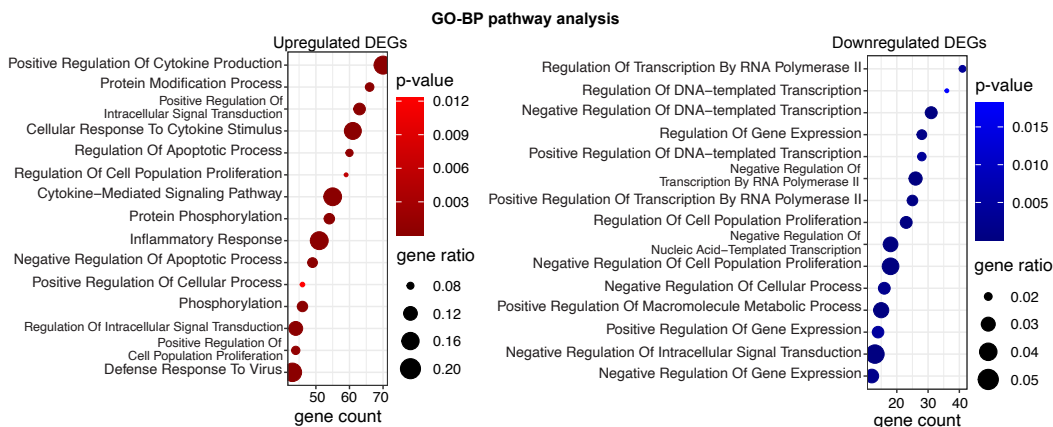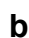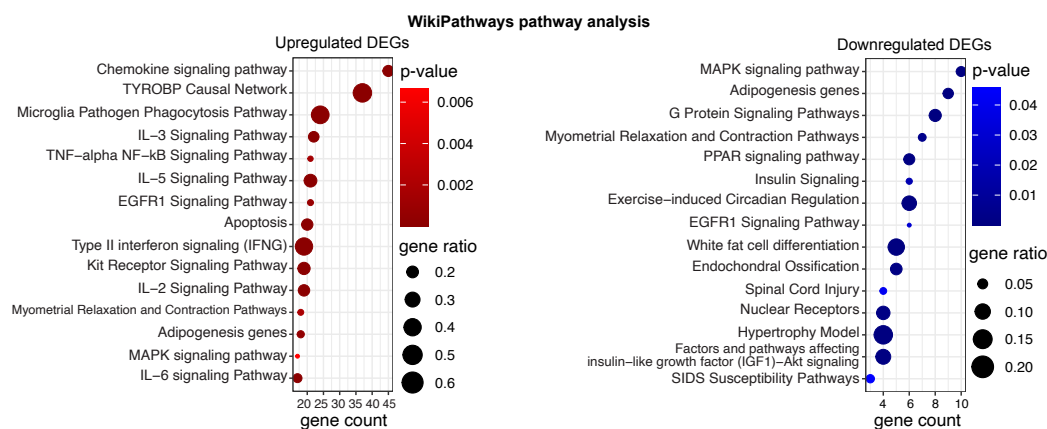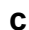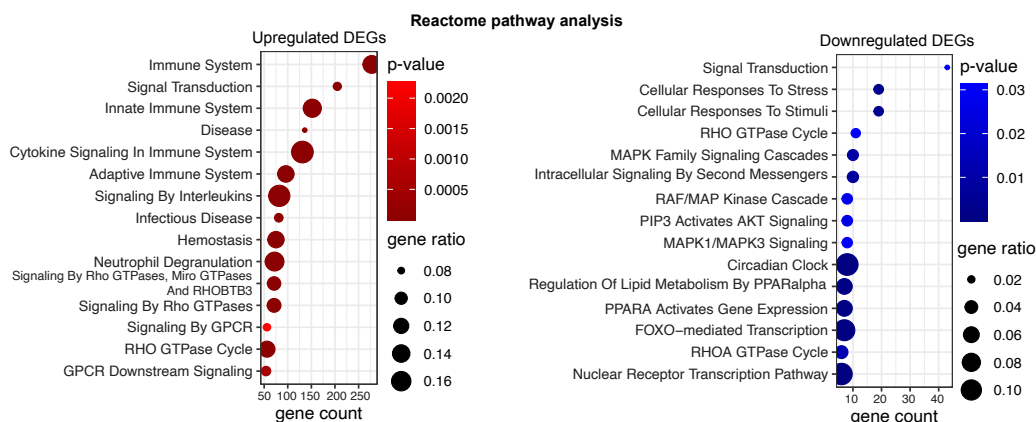

**a**

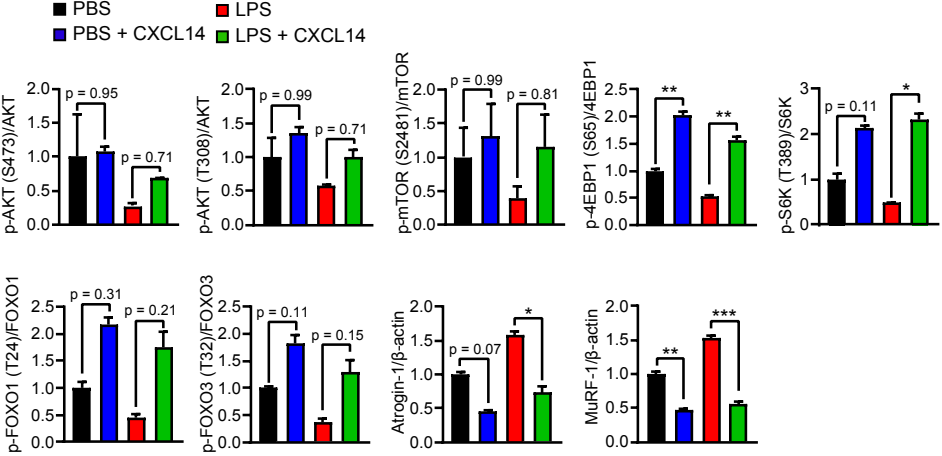

**b**

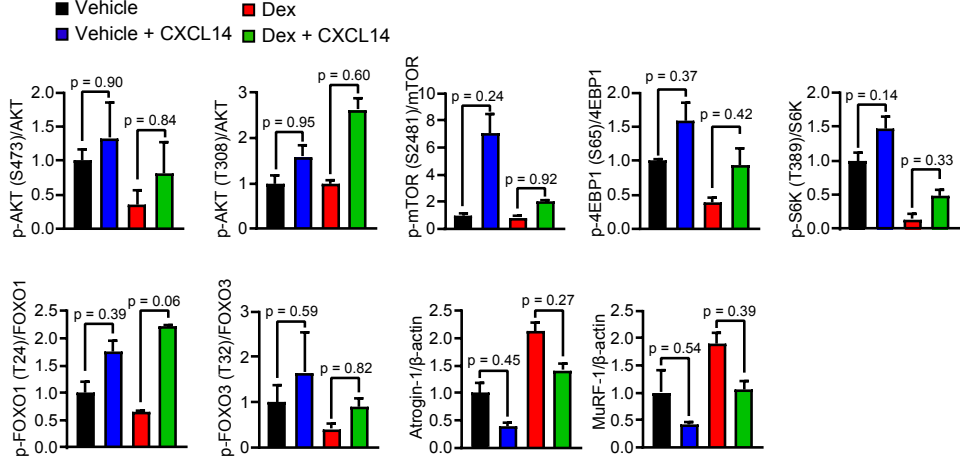

Fig. S7

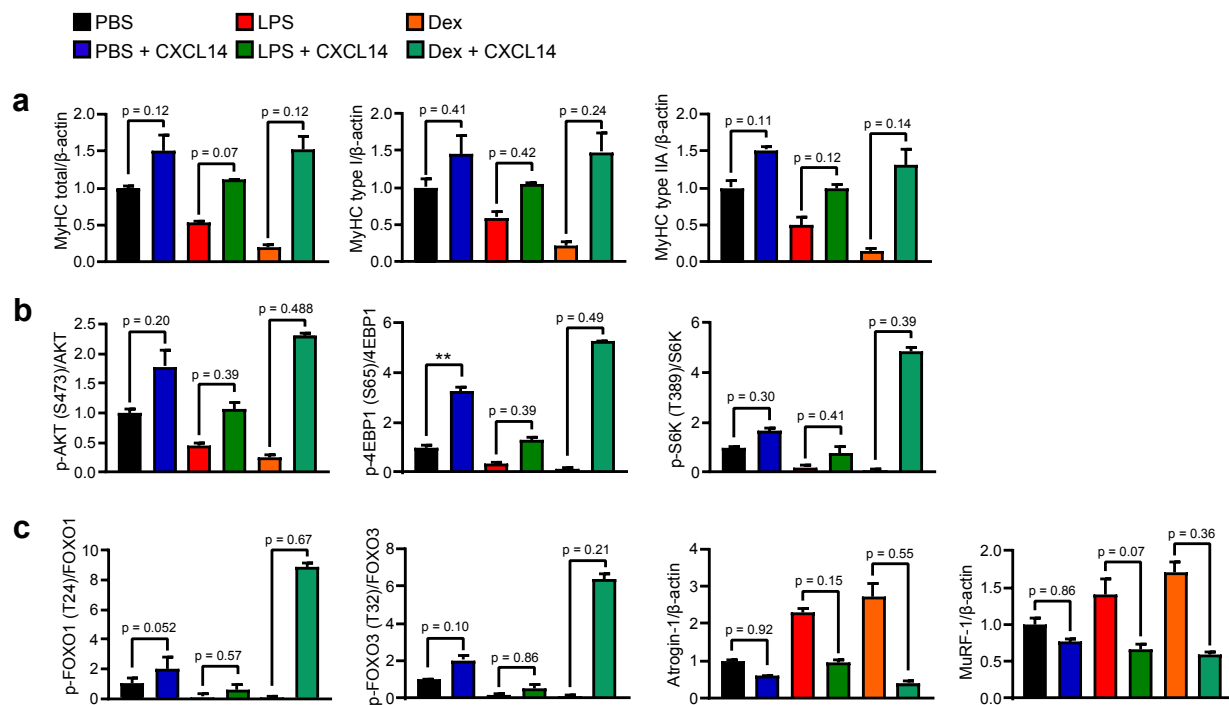

Fig. S8

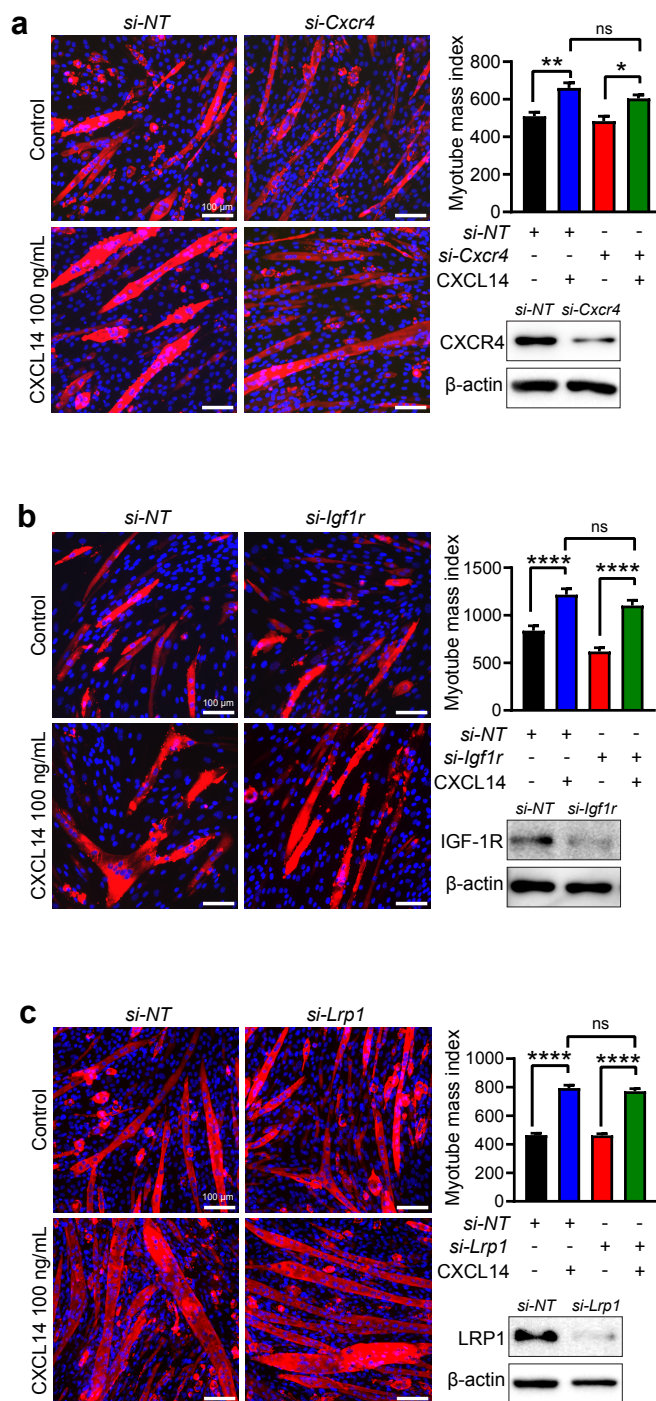

Supplement: Supplementary file 3 — Table S2: Effects of CXCL14 on myogenic differentiation in C2C12 cells. Table S3: Mass index of C2C12 myotubes treated with CXCL14 in differentiation medium. Table S4: Effect of Rps6kb1 gene knockdown on C2C12 myotube mass. Table S5: Effect of Cxcl14 overexpression in cross‐sectional area (CSA) of TA muscle. Table S6: Effect of Cxcl14 overexpression on cross‐sectional area (CSA) of different fibre types in TA muscle. Table S7: Mass index of LPS‐treated C2C12 myotubes. Table S8: Effect of Cxcl14 overexpression on TA muscle cross‐sectional area (CSA) in LPS‐treated mice. Table S9: Mass index of dexamethasone‐treated C2C12 myotubes. Table S10: Effect of Cxcl14 overexpression on TA muscle cross‐sectional area (CSA) in dexamethasone (Dex)‐treated mice TA muscle. Table S11: Effect of CXCL14 on mass index of primary human myotubes treated with LPS or dexamethasone. Table S12: Effect of putative CXCL14 receptor gene knockdowns on C2C12 myotube mass index. [file JCSM-16-e70087-s004.pdf]
