# Supplementary material for: CXCL14 Promotes Skeletal Muscle Mass Growth and Attenuates Lipopolysaccharide‐ and Dexamethasone‐Induced Muscle Atrophy in Cultured Myotubes and Mouse Models
Source: J Cachexia Sarcopenia Muscle. 2025 Oct 14;16(5):e70087. doi: 10.1002/jcsm.70087 (PMC12519514; doi:10.1002/jcsm.70087)
Supplement: Supplementary file 4 — Figure S1: jcsm70087‐sup‐0004‐Supplementary_Legends.docx. No effect of CXCL14 on myogenic differentiation in vitro. (a) C2C12 cells were differentiated for 2 days with or without recombinant CXCL14 protein (20 and 100 ng/mL). Myogenin (MyoG) protein expression was assessed by immunofluorescence staining with an anti‐MyoG antibody (green), while DAPI (blue) was employed for nuclear counterstaining. Scale bar = 100 μm. The differentiation index, defined as the percentage of MyoG‐positive nuclei over total nuclei, was calculated from more than 300 cells. (b) C2C12 cells were differentiated for 4 days with or without recombinant CXCL14 protein (20 and 100 ng/mL). MyHC protein expression was assessed by immunofluorescence staining with an anti‐MyHC antibody (red), with DAPI (blue) used for nuclear counterstaining. Scale bar = 100 μm. The differentiation index and fusion index (distribution of myocytes (mononuclear) and myotubes (2–4 nuclei and > 5 nuclei) in MyHC‐positive cells) were calculated from over 300 cells. (c) Western blot analysis of myogenic differentiation markers was performed, with CXCL14‐treated and control cells harvested at four time points (1, 2, 3, 4 days of differentiation). Quantification of the Western blot data is presented in the right panels. (d) C2C12 myotubes obtained after 4 days of differentiation treated with recombinant CXCL14 protein (20 and 100 ng/mL) for two days in differentiation medium. MyHC protein expression was assessed by immunofluorescence staining with an anti‐MyHC antibody (red), with DAPI (blue) used for nuclear counterstaining. Scale bar = 100 μm. Myotube mass indices (MMIs) were calculated as the area of MyHC‐positive myotube divided by the number of nuclei (only myotubes with more than seven nuclei were counted). (e) Western blot analysis of AKT and FOXO proteins expression in CXCL14‐treated and control myotubes. C2C12 myotubes were treated with CXCL14 for 2 days in differentiation medium. Quantification of the Western blot [file JCSM-16-e70087-s003.docx]

**Supplementary figure legends**

**Supplementary Figure 1. No effect of CXCL14 on myogenic differentiation *in vitro*.** (a) C2C12 cells were differentiated for 2 days with or without recombinant CXCL14 protein (20 and 100 ng/mL). Myogenin (MyoG) protein expression was assessed by immunofluorescence staining with an anti-MyoG antibody (green), while DAPI (blue) was employed for nuclear counterstaining. Scale bar = 100 μm. The differentiation index, defined as the percentage of MyoG-positive nuclei over total nuclei, was calculated from more than 300 cells. (b) C2C12 cells were differentiated for 4 days with or without recombinant CXCL14 protein (20 and 100 ng/mL). MyHC protein expression was assessed by immunofluorescence staining with an anti-MyHC antibody (red), with DAPI (blue) used for nuclear counterstaining. Scale bar = 100 μm. The differentiation index and fusion index (distribution of myocytes (mononuclear) and myotubes (2-4 nuclei and >5 nuclei) in MyHC-positive cells) were calculated from over 300 cells. (c) Western blot analysis of myogenic differentiation markers was performed, with CXCL14-treated and control cells harvested at four time points (1, 2, 3, 4 days of differentiation). Quantification of the Western blot data is presented in the right panels. (d) C2C12 myotubes obtained after 4 days of differentiation treated with recombinant CXCL14 protein (20 and 100 ng/mL) for two days in differentiation medium. MyHC protein expression was assessed by immunofluorescence staining with an anti-MyHC antibody (red), with DAPI (blue) used for nuclear counterstaining. Scale bar = 100 μm. Myotube mass indices (MMIs) were calculated as the area of MyHC-positive myotube divided by the number of nuclei (only myotubes with more than seven nuclei were counted). (e) Western blot analysis of AKT and FOXO proteins expression in CXCL14-treated and control myotubes. C2C12 myotubes were treated with CXCL14 for 2 days in differentiation medium. Quantification of the Western blot data is presented in the right panels. Data are expressed as mean ± SEM. To calculate p-values, one-way ANOVA with Tukey’s post hoc test was used for panels (a), (b), (d), and (e), while an unpaired student’s t-test was applied for panel (c). *p ≤ 0.05; **p ≤ 0.01; ***p ≤ 0.001

**Supplementary Figure 2. Quantification of Western blot data following CXCL14 treatment.** (a-b) Relative fold change in MyHC isoform expression (a), and phosphorylation of AKT-S6K and FOXO1/3 pathway components (b) in myotubes treated with CXCL14 for 48 hours. (c) Western blot analysis of AKT-S6K and FOXO pathways in myotubes treated with CXCL14 for up to 2 h at 100 ng/mL (left). Relative fold change in phosphorylation of the AKT-S6K (middle) and FOXO1/3 (right) pathway components over a time course of CXCL14 treatment. (d) Relative fold change in MyHC isoform expression in *Rps6kb1* knockdown C2C12-derived myofibers treated with CXCL14. Data are presented as mean ± SEM. To calculate p-values, one-way ANOVA with Tukey’s post hoc test was used for panels (a), (b) and (d), while repeated measures ANOVA with Dunnett’s post hoc test was applied for panel (c). *p ≤ 0.05; **p ≤ 0.01; ***p ≤ 0.001; ****p ≤ 0.0001

**Supplementary Figure 3.** ***Cxcl14* overexpression does not affect myofiber type determination**. (a) TA muscle sections were immunofluorescently stained for the expression of Laminin (green) and various MyHC isoforms (red). All images were captured in at least five randomly selected fields. Scale bar = 50 μm. (b) Percentage of different myofiber types relative to the total number of myofibers. (c) Distribution of CSA of different myofiber type in control (black), CXCL14-Myc (blue), and HA-CXCL14 (green) overexpressed TA muscles are presented on the left. Average and median CSA are shown on the right. Data are expressed as mean ± SEM (C and D). To calculate p-values, one-way ANOVA with Tukey’s post hoc test was used for average CSA, while Kruskal-Wallis with Dunn’s post hoc test was applied to median CSA. *p ≤ 0.05; **p ≤ 0.01; ***p ≤ 0.001; ****p ≤ 0.0001

**Supplementary Figure 4. Transcriptome analysis of *Cxcl14*-overexpressed TA muscles.** (a) Workflow for transcriptome analysis. (b) Volcano plot of DEGs. Blue and red dots represent downregulated and upregulated DEGs, respectively.

**Supplementary Figure 5. Annotation analyses with DEGs in *Cxcl14*-overexpressed TA muscles.** (a) Top 15 GO-BP terms enriched in upregulated (red) and downregulated (blue) DEGs. (b) Top 15 WikiPathways terms enriched in upregulated (red) and downregulated (blue) DEGs. (c) Top 15 Reactome terms enriched in upregulated (red) and downregulated (blue) DEGs.

**Supplementary Figure 6. Quantification of Western blot data from LPS- or DEX-induced atrophy restored by CXCL14 in C2C12-derived myotubes.** Relative fold change in the expression of AKT-S6K and FOXO1/3 pathway components in (a) LPS- and (b) DEX-induced atrophic myotubes, with or without CXCL14. Data are presented as mean ± SEM. One-way ANOVA with Tukey’s post hoc test was used for p-value calculation. *p ≤ 0.05; **p ≤ 0.01; ***p ≤ 0.001; ****p ≤ 0.0001

**Supplementary Figure 7. Quantification of Western blot data from LPS- and DEX-treated primary human muscle cells.** Relative fold change in the expression of different (a) MyHC isoforms, along with components of the (b) AKT-S6K and (c) FOXO1/3 pathways, in LPS- and DEX-treated myotubes, with or without CXCL14. Data are expressed as mean ± SEM. One-way ANOVA with Tukey’s post hoc test was used to calculate p-values. *p ≤ 0.05; **p ≤ 0.01; ***p ≤ 0.001; ****p ≤ 0.0001

**Supplementary Figure 8. CXCL14-induced hypertrophy is independent of known CXCL14 receptors.** C2C12 cell-derived myotubes were transfected with siRNAs [si-NT(*NonTarget)*, si-*Cxcr4*, si-*Igf1r*, or si-*Lrp1*] and treated with CXCL14. Myotubes were stained with anti-MyHC antibody (red) and counterstained with DAPI (blue) for nuclei to determine MMI. Scale bar = 100 μm. Gene knockdown efficiency by siRNAs was determined by Western blot analysis. Data are presented as mean ± SEM. One-way ANOVA with Tukey’s post hoc test was used to calculate p-values. *p ≤ 0.05; **p ≤ 0.01; ***p ≤ 0.001; ****p ≤ 0.0001
